# Glycogen availability and pH variation in a medium simulating vaginal fluid influence the growth of vaginal *Lactobacillus* species and *Gardnerella vaginalis*

**DOI:** 10.1186/s12866-023-02916-8

**Published:** 2023-07-13

**Authors:** Stephany Navarro, Habib Abla, Betsaida Delgado, Jane A. Colmer-Hamood, Gary Ventolini, Abdul N. Hamood

**Affiliations:** 1 Department of Immunology and Molecular Microbiology, Texas Tech University Health Sciences Center, Lubbock, TX USA; 2 School of Medicine, Texas Tech University Health Sciences Center, Lubbock, TX USA; 3 Honors College, Texas Tech University, Lubbock, TX USA; 4 Department of Medical Education, Texas Tech University Health Sciences Center, Lubbock, TX USA; 5 Department of Obstetrics and Gynecology, Texas Tech University Health Sciences Center Permian Basin, Odessa, TX USA; 6 Department of Surgery, Texas Tech University Health Sciences Center, Lubbock, TX USA; 7 Woody L. Hunt School of Dental Medicine, Texas Tech University Health Sciences Center, Lubbock, TX USA

**Keywords:** *Lactobacillus jensenii*, *Lactobacillus gasseri*, *Lactobacillus crispatus*, *Gardnerella vaginalis*, Medium simulating vaginal fluid, pH, Glycogen, Glucose

## Abstract

**Background:**

Glycogen metabolism by *Lactobacillus* spp. that dominate the healthy vaginal microbiome contributes to a low vaginal pH (3.5–4.5). During bacterial vaginosis (BV), strict and facultative anaerobes including *Gardnerella vaginalis* become predominant, leading to an increase in the vaginal pH (> 4.5). BV enhances the risk of obstetrical complications, acquisition of sexually transmitted infections, and cervical cancer. Factors critical for the maintenance of the healthy vaginal microbiome or the transition to the BV microbiome are not well defined. Vaginal pH may affect glycogen metabolism by the vaginal microflora, thus influencing the shift in the vaginal microbiome.

**Results:**

The medium simulating vaginal fluid (MSVF) supported growth of *L. jensenii* 62G, *L. gasseri* 63 AM, and *L. crispatus* JV-V01, and *G. vaginalis* JCP8151A at specific initial pH conditions for 30 d. *L. jensenii* at all three starting pH levels (pH 4.0, 4.5, and 5.0), *G. vaginalis* at pH 4.5 and 5.0, and *L. gasseri* at pH 5.0 exhibited the long-term stationary phase when grown in MSVF. *L. gasseri* at pH 4.5 and *L. crispatus* at pH 5.0 displayed an extended lag phase over 30 d suggesting inefficient glycogen metabolism. Glycogen was essential for the growth of *L. jensenii, L. crispatus*, and *G. vaginalis*; only *L. gasseri* was able to survive in MSVF without glycogen, and only at pH 5.0, where it used glucose. All four species were able to survive for 15 d in MSVF with half the glycogen content but only at specific starting pH levels – pH 4.5 and 5.0 for *L. jensenii*, *L. gasseri*, and *G. vaginalis* and pH 5.0 for *L. crispatus*.

**Conclusions:**

These results suggest that variations in the vaginal pH critically influence the colonization of the vaginal tract by lactobacilli and *G. vaginalis* JCP8151A by affecting their ability to metabolize glycogen. Further, we found that *L. jensenii* 62G is capable of glycogen metabolism over a broader pH range (4.0–5.0) while *L. crispatus* JV-V01 glycogen utilization is pH sensitive (only functional at pH 5.0). Finally, our results showed that *G. vaginalis* JCP8151A can colonize the vaginal tract for an extended period as long as the pH remains at 4.5 or above.

**Supplementary Information:**

The online version contains supplementary material available at 10.1186/s12866-023-02916-8.

## Background

The two main features of the vaginal ecosystem of a healthy reproductive-age woman are an acidic environment with a pH range of 3.8 to 4.2 and the presence of several types of antimicrobial molecules including mucins, antibodies, and β-defensins [1–4]. Additionally, the vaginal microbiome is dominated by four acid-producing *Lactobacillus* spp. – *L. jensenii*, *L. crispatus*, *L. gasseri*, and *L. iners* [2, 4]. Due to their ability to produce considerable levels of lactic acid, colonization of the vaginal mucosa with these lactobacillus helps maintains the low pH, which may reach as low as 3.5 [2, 5, 6]. Besides lowering the pH, these lactobacilli protect the vagina and promote vaginal health through the production of other factors including hydrogen peroxide, bacteriocins, and biosurfactants as well as adhesins that allow the lactobacilli to competitively exclude other bacteria [7–9]. Loss of the protective effect of the lactobacilli produces a dysbiotic shift in the vaginal microbiome known as bacterial vaginosis (BV), which is characterized by the overgrowth of anaerobic and facultatively anaerobic bacteria, especially *Gardnerella vaginalis* [2, 10]. Symptomatic BV is indicated by an increase in the vaginal pH, malodorous discharge, dysuria, dyspareunia, and vaginal pruritus [5, 11]. Bacterial vaginosis is also associated with several adverse conditions including an increased risk for a pre-term birth [12], acquisition of sexually transmitted infections, including human immunodeficiency virus, *Neisseria gonorrhoeae*, *Chlamydia trachomatis*, and *Trichomonas* spp [13–16]., and human papillomavirus and cancer [17, 18].

Pyrosequencing analysis of samples obtained from asymptomatic North American women with different ethnic backgrounds revealed clustering of the vaginal microbial communities of species, which came to be called community state types (CST); four of which were dominated by one of the *Lactobacillus* spp. – *L. crispatus* (I), *L. gasseri* (II), *L. iners* (III), or *L. jensenii* (V), and one containing high proportions of strictly anaerobic bacteria and *G. vaginalis* (IV) [2, 19]. As expected, the highest average pH (5.3) was observed with CST IV, which was most common in Black and Hispanic women; many of whom, but not all, met Nugent’s criteria for BV [2]. The lowest average pH (4.0) was observed with CST I, which was more common in White women, none of whom had BV by Nugent’s criteria [2]. Therefore, Ravel et al. suggested that a low pH and a *Lactobacillus-*dominated population are not completely indicative of a woman being “healthy” and that what is a “normal” vaginal microflora may be different depending on the racial/ethnic background of the individual [2]. Gajer et al. found that the community groups are not static but dynamic, with shifts from one state type to another for short periods of time [20]. Later, Ma et al. reported that, even within a cluster dominated by a single *Lactobacillus* sp., significant intraspecies genetic variations were detected [21].

Glycogen is a large energy storage molecule consisting of ⍺- and β-chains with about 13 glucose residues each. The ⍺-chains are covalently linked through ⍺-1,4-glycosidic bonds to form non-branched chains and β-chains on the other hand, are attached through ⍺-1,6-glucosidic bonds to form branched chains [22–24]. These ⍺- and β-chains are linked together in tiers, consisting of about 53,000 glucose residues, to make one glycogen molecule [23, 24]. At the onset of puberty, glycogen deposition and accumulation in the cervical and vaginal epithelial cells coincide with the increase in the level of circulating estrogen [25, 26]. Glycogen stored within the vaginal epithelial cells is converted to glucose, which is then metabolized under anaerobic conditions to produce energy; a process that eventually produces lactic acid that diffuses from the epithelial cells and accumulates within the vaginal fluid [7, 27]. When the vaginal epithelial cells are sloughed off and lysed by various vaginal bacteria, glycogen also accumulates extracellularly within the vaginal canal at concentrations ranging from 0.1 to 32 µg/mL [27]. Glycogen is metabolized by the host enzyme ⍺-amylase, an extracellular glycoside hydrolase that preferentially cleaves the ⍺-1,4-glycosidic bonds within glycogen, as well as ⍺-dextrin molecules that contain both ⍺-1,6-linked branches and ⍺-1,4-linkages, producing maltose and maltotriose that may be utilized by bacterial amylopullulanase [28, 29]. Metabolism of the extracellular glycogen as well as glucose fermentation produce lactic acid, which lowers the pH of the vaginal environment and prevents BV-associated bacteria from overpopulating the vaginal mucosa and maintains vaginal health [6, 9, 16].

Two of the most widely used media for research with *Lactobacillus* spp. and *G. vaginalis* are de Man, Rogosa, and Sharpe broth (MRSB) and New York City III broth (NYCB), respectively [30, 31]. Both MRSB and NYCB contain carbohydrates, amino acids, vitamins, and cofactors to support the complex nutritional requirements of these organisms [30–33]. However, despite the presence of these nutritional requirements, neither medium closely reflects the components of the vaginal fluid. Vaginal fluid consists of transudate from the vaginal epithelium, secretions from glands near the vaginal opening, cervical mucus, and fluids from the endometrium and uterine tubes [34]. While consisting mainly of water (95%), vaginal fluid includes salts, urea, carbohydrate, mucin, albumin, fatty acids, and immunoglobulins [34]. Several artificial media that contain different components of the vaginal fluid have been described including artificial vaginal fluid [35], the chemically defined medium [36], vaginal fluid simulant [37], medium simulating vaginal fluid (MSVF) [38], and vaginal fluid simulants with and without glucose [39]. Among these media, MSVF was designed to test the in vitro growth, pH modifications, and expression of beneficial characteristics of lactobacilli [38].

As described above, changes in the vaginal pH significantly impact the transition in the vaginal state from a healthy to a diseased one by influencing the vaginal microflora. A lower pH favors the growth of vaginal lactobacilli and facilitates their ability to colonize the vaginal epithelium thereby producing a healthy vaginal environment. In contrast, an increase in the vaginal pH supports the growth of vaginal pathogens such as *G. vaginalis* and enhances their ability to outcompete the vaginal lactobacilli in colonizing the vaginal epithelium, thereby producing BV. Changes in vaginal pH likely accomplish these effects by influencing the metabolism of vaginal microflora. Despite numerous previous studies, the influence of pH changes on the growth of vaginal lactobacilli or *G. vaginalis* under conditions mimicking the vaginal environment has not been thoroughly analyzed. In this study we conducted a detailed and extended growth curve (30 d) analysis of three of the vaginal lactobacilli (*L. jensenii* 62G, *L. gasseri* 63 AM, and *L. crispatus* JV-V01) and *G. vaginalis* JCP8151A individually under three specific pH conditions (4.0, 4.5, and 5.0) using MSVF, which closely mimics vaginal fluid, compared to standard laboratory media used for the growth of these bacteria. We also explored the effect of these pH conditions on the metabolism of these bacteria; specifically, their ability to metabolize glycogen.

## Results

### The type of medium and starting pH affect growth of the ***Lactobacillus*** spp. and ***G. vaginalis***

#### G. vaginalis ***JCP8151A grew for 30 d at pH 4.5 and 5.0 in MSVF***

**Fig. 1:**
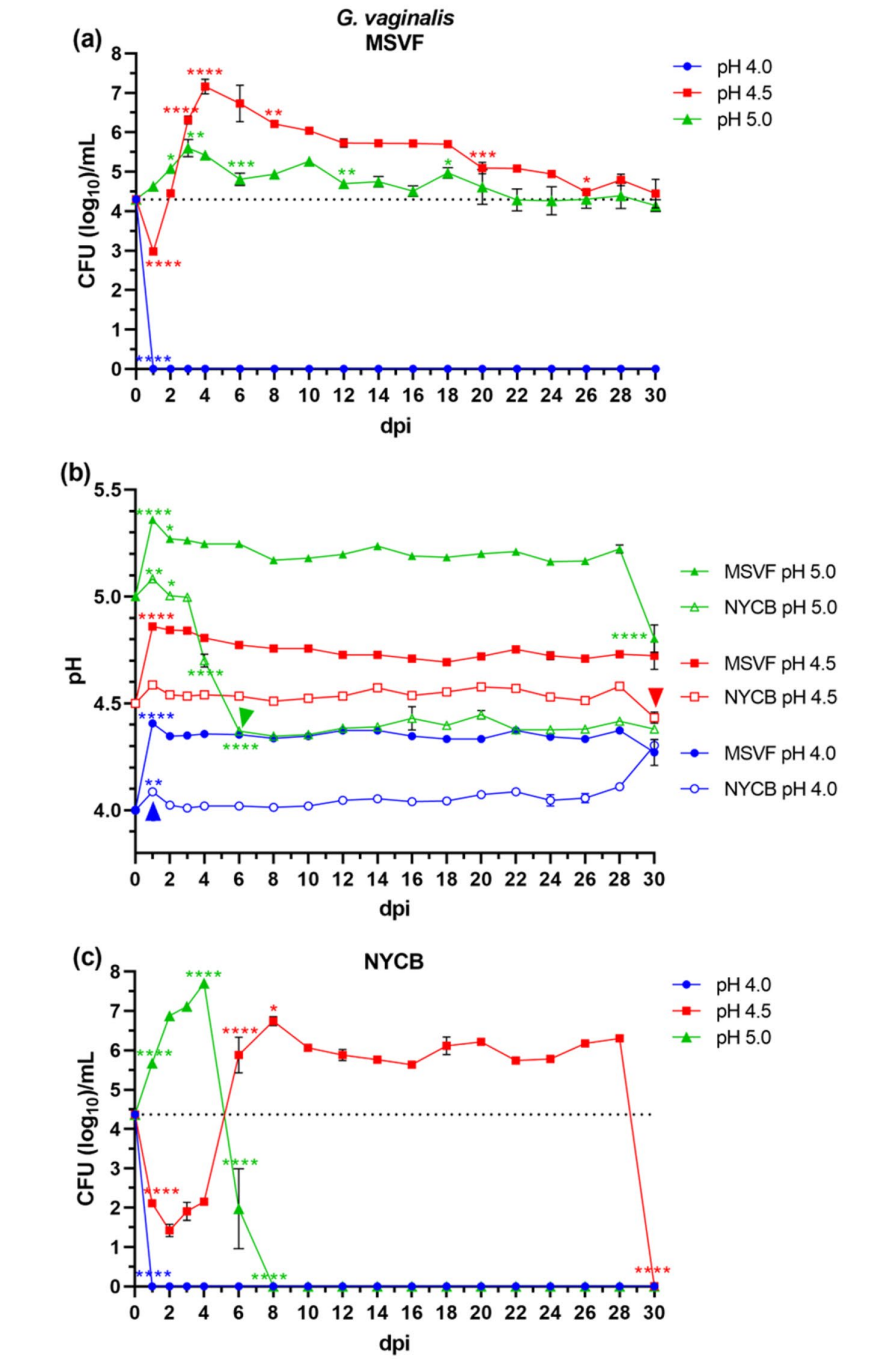
Growth patterns and changes in pH of *G. vaginalis* JCP8151A grown in MSVF and NYCB. *G. vaginalis* was grown overnight in NYCB at pH 7.3; cells were pelleted, washed, and resuspended in MSVF at pH 4.0, pH 4.5, and pH 5.0. One-mL aliquots were pipetted into the wells of a 24-well microtiter plate and inoculated with ~ 10^4^ CFU. The plates were sealed with breathable membrane and the cultures were incubated at 37 °C under 5% CO_2_ in a humid chamber for 30 dpi. Samples were taken at 1-d intervals through 4 dpi and then every 2 d over the 30-d growth cycle and the CFU/mL and pH were determined. **(a)** Viability of *G. vaginalis* grown in MSVF for 30 d at starting pH of 4.0, 4.5, and 5.0; **(b)** pH of MSVF and NYCB at each time point throughout the growth cycle; **(c)** viability of *G. vaginalis* grown in NYCB for 30 d at starting pH of 4.0, 4.5, and 5.0. Each symbol represents the mean of three independent experiments ± SOM. Dotted lines (a, c) indicate starting CFU/mL. Arrowheads (b) indicate loss of viability. Two-way ANOVA with Tukey’s multiple comparisons posttest was done to determine significant differences between time points across the growth curves. *, *P* < 0.05; **, *P* < 0.01; ***, *P* < 0.001; ****, *P* < 0.0001

There was no growth of *G. vaginalis* JCP8151A under starting pH condition of 4.0; however, although within the first day the initial inoculum of 10^4^ was reduced to 10^3^, the growth at pH 4.5 did not terminate (Fig. 1a). This lag phase of the growth curve represents the time necessary for bacteria to adjust to the stress of the new environmental conditions [40]. Unlike the growth at pH 4.0, this reduction did not continue. Rather, the growth increased from 10^3^ to 10^7^ within 4 d (1–5 days post inoculum [dpi]) (Fig. 1a). This likely represents the exponential phase of growth. From d 5 until d 30, there was a gradual decline in the growth. This likely represents a stationary phase which is considerably longer compared with the 16–24 h stationary phase of bacterial growth in nutrient-rich medium. The decline in the growth within this entire 22-d stationary phase resulted in a two-log reduction (10^7^ to 10^5^) (Fig. 1a). The main feature of the growth under the starting pH condition of 5.0 was the absence of any reduction (Fig. 1a). Like the growth under pH 4.5, there appeared to be a short exponential phase in which the initial inoculum increased to 10^5^ CFU/mL (Fig. 1a). This was followed by a prolonged stationary phase also known as the long-term stationary phase (LTSP) in which there was limited or no reduction in the growth (Fig. 1a). Maintenance of this viability does not represent a starvation mode induced by a severe limitation of the nutrients, as has been described from other bacteria [41]. Rather than the slow and gradual reduction of growth throughout this prolonged period, starvation of the bacteria would have likely been preceded by a significant reduction of growth.

Under pH conditions of 4.5 and 5.0, the pH of the cultures increased at d 1 where they remained stable for the course of the experiment, except for a drop in pH from 5.2 to 4.8 at 30 dpi in MSVF at starting pH 5.0 (Fig. 1b). At pH 4.5, changes in pH roughly followed changes in CFU; at pH 5.0, the drop in pH of the culture from 5.2 to 4.8 was associated with a small drop in bacterial viability (Fig. S1a, b).

#### G. vaginalis JCP8151A also grew at pH 4.5 and 5.0 in NYCB but not for the full 30 d

At a starting pH of 4.0, the growth pattern was like that in MSVF where the growth terminated in one day (Fig. 1c), At a starting pH of 4.5, the pattern was similar to that in MSVF with the following unique features: at 2 dpi, the reduction of about three logs (from 10^4^ to 10^1^), was more prominent than that in MSVF; this was followed by an exponential phase with a sharp increase between 4 and 8 dpi (10^1^ to 10^7^); between 8 and 28 dpi, there was an apparent stationary phase in which *G. vaginalis* maintained viability without significant changes in growth; immediately after d 28, the growth was terminated as there were no recoverable CFU at 30 dpi (Fig. 1c). Currently, the reason for the sudden loss of growth is not known. It is possible that it is related to pH as the pH dropped from 4.6 to 4.4 concomitant with loss of viability (Fig. S2a). We do not suspect a depletion of nutrients or starvation as a reduction caused by these instances would be gradual and not abrupt. At a starting pH of 5.0, and like that in MSVF, there was no initial reduction in growth (Fig. 1c). Compared to MSVF, the exponential phase was more prominent with an increase of about three logs within the first 4 dpi (Fig. 1c). However, in contrast to MSVF, and immediately following the peak of 10^7^ CFU/mL, bacterial viability was lost (0 CFU at 8 dpi) (Fig. 1c).

Concordant with the sudden loss of viability, there was a change in the pH of the culture from 5.0 to 4.4 between 3 and 6 dpi (Fig. 1b, S2b). As seen with the loss of viability in NYCB pH 4.5, it is possible that a pH below 4.5 is outside of the range for viability of *G. vaginalis*.

#### L. jensenii 62G grew for 30 d at all three starting pH ranges in MSVF

**Fig. 2:**
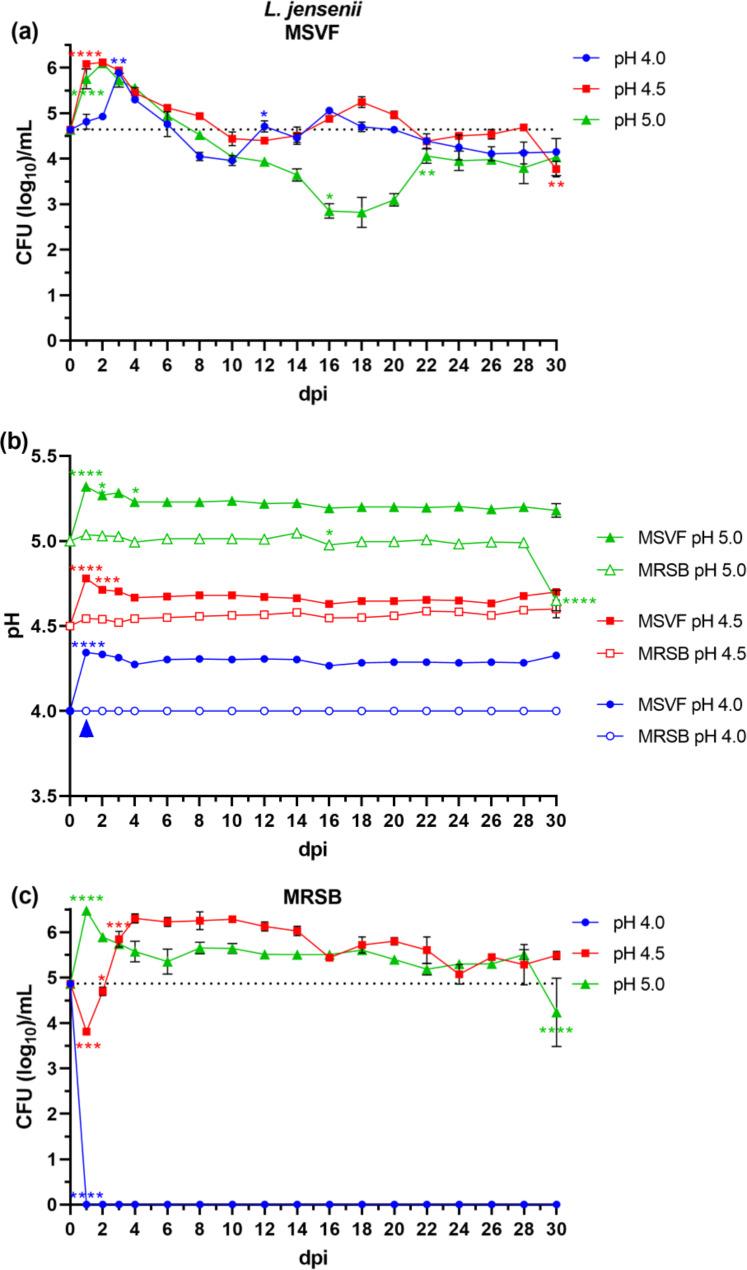
Growth patterns and changes in pH of *L. jensenii* 62G grown in MSVF and MRSB. *L. jensenii* was grown overnight in MRSB at pH 6.3 and processed as described in Fig. 1. **(a)** Viability of *L. jensenii* grown in MSVF for 30 d at starting pH of 4.0, 4.5, and 5.0; **(b)** pH of MSVF and MRSB at each time point throughout the growth cycle; **(c)** viability of *L. jensenii* grown in MRSB for 30 d at starting pH of 4.0, 4.5, and 5.0. Each symbol represents the mean of three independent experiments ± SOM. Dotted lines (a, c) indicate starting CFU/mL. Arrowheads (b) indicate loss of viability. Two-way ANOVA with Tukey’s multiple comparisons posttest was done to determine significant differences between time points across the growth curves. *, *P* < 0.05; **, *P* < 0.01; ***, *P* < 0.001; ****, *P* < 0.0001

The growth of *L. jensenii* 62G in MSVF was unique in that unlike *G. vaginalis*, *L. jensenii* grew under a starting pH of 4.0 (Fig. 2a). This growth was maintained until the end of the 30-d growth period and except for some slight variations, the pattern of growth under all three starting pH conditions remained similar, that is, the LTSP (Fig. 2a). The growth cycle started with an initial increase in growth within 2 dpi under pH conditions of 4.5 and 5.0 (about 1.5 logs) (Fig. 2a). This initial increase was slightly delayed until d 3 under a pH of 4.0 (Fig. 2a). Under all three pH conditions, the increase was followed by a gradual decline until about 10 dpi (Fig. 2a). Under pH 4.0 the decline in growth stops at 4 logs, after which the growth of *L. jensenii* increased and decreased over a one-log interval (Fig. 2a). This cyclic pattern was also observed under a pH condition of 4.5 where the decline in growth stopped at 4.5 logs and increased and decreased again by one log (Fig. 2a). A decline in growth, which was more prominent, continued until 16 dpi (3-log decrease) (Fig. 2a). Over the course of the next 6 d (16–22 dpi), the growth was increased by one log (Fig. 2a). From 22 dpi onward, and like growth curves under pH conditions of 4.0 and 4.5, *L. jensenii* maintained viability until the end of the cycle (Fig. 2a).

Under starting pH conditions of 4.5 and 5.0 and except for the initial increase at 1 dpi, the pH remained constant across the 30-d growth cycle (Fig. 2b). Only at pH 4.5 were the CFU similar to the pH levels (Fig. S1c-e).

#### L. jensenii 62G grew for 30 d at pH 4.5 and 5.0 in MRSB

Similar to the growth pattern of *G. vaginalis* in NYCB at 1 dpi, we obtained no viable *L. jensenii* 62G colonies under starting condition of pH 4.0 (Fig. 2c). Under a starting pH of 4.5, there was an initial one-log reduction at 1 dpi followed by an exponential phase (3 log increase) until 4 dpi (Fig. 2c). Starting from 4 dpi and through 30 dpi, *L. jensenii* maintained viability with no significant increase or decrease in growth (Fig. 2c). The growth pattern under a pH of 5.0 was similar to that of pH 4.5 in MSVF, except for a one-log drop in CFU at 30 dpi (Fig. 2c).

The growth in MRSB under pH 4.5 and 5.0 conditions was associated with little change in the pH except for a drop in the culture under pH conditions of 5.0 from 5.0 to 4.6 at 30 dpi that correlated with the drop in CFU (Fig. 2b, S2c,d).

#### L. gasseri 63 AM also grew for 30 d at pH 4.5 and 5.0 in MSVF

**Fig. 3:**
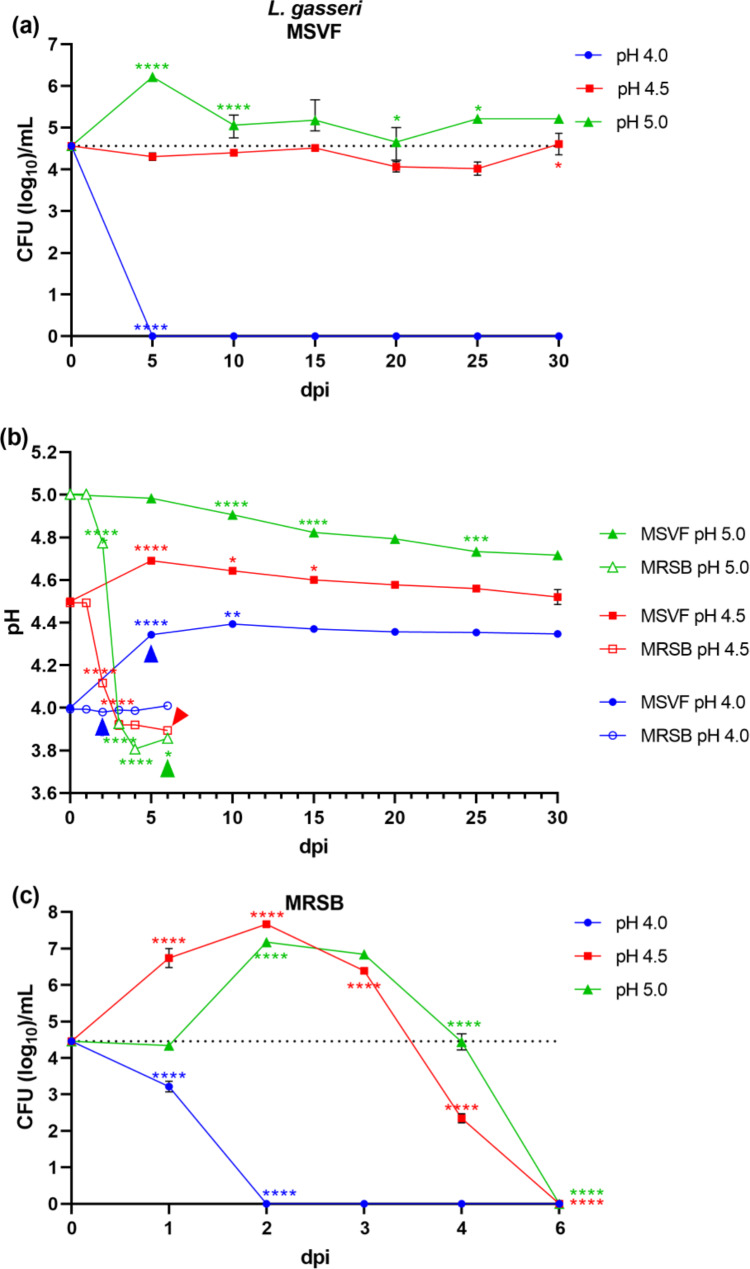
Growth patterns and changes in pH of *L. gasseri* 63 AM grown in MSVF and MRSB. *L. gasseri* was grown overnight in MRSB at pH 6.3 and processed as described in Fig. 1. **(a)** Viability of *L. gasseri* grown in MSVF for 30 d at starting pH of 4.0, 4.5, and 5.0; **(b)** pH of MSVF and MRSB at each time point throughout the growth cycle; **(c)** viability of *L. gasseri* grown in MRSB for 30 d at starting pH of 4.0, 4.5, and 5.0. Each symbol represents the mean of three independent experiments ± SOM. Dotted lines (a, c) indicate starting CFU/mL. Arrowheads (b) indicate loss of viability. Two-way ANOVA with Tukey’s multiple comparisons posttest was done to determine significant differences between time points across the growth curves. *, *P* < 0.05; ***, *P* < 0.001; ****, *P* < 0.0001

Unlike the growth curve of *G. vaginalis* and *L. jensenii*, where we obtained samples every 2 d, we obtained samples every 5 d throughout the 30-d period. Under starting pH conditions of 4.0, and unlike *L. jensenii*, we detected no viable *L. gasseri* 63 AM CFU at 5 dpi (Fig. 3a). Also unlike the growth of *L. jensenii* and more like the growth of *G. vaginalis* under these conditions, the loss of viable CFU likely occurred earlier at 1 or 2 dpi (Fig. 3a). The growth pattern in MSVF at pH 4.5 was flat, characteristic of an extended lag phase that continued for 30 dpi while the growth pattern in MSVF at pH5.0 resembled the LTSP and continued for 30 dpi (Fig. 3a).

The pH of the culture at pH 4.5 reached a high of 4.7 at 5 dpi before dropping back to 4.5 at 30 dpi while the pH of the MSVF culture at 5.0 dropped steadily over the 30-d cycle to a low of 4.7 (Fig. 3b). No correlation in pH and CFU was noted (Fig. S1f, g).

#### L. gasseri 63 AM lost viability by 5 dpi in MRSB at pH 4.5 and 5.0

Under starting pH conditions of 4.0, the number of CFU declined by more than 1 log on d 1 and was terminated by 2 dpi (Fig. 3c). Under pH starting conditions of 4.5 and 5.0, the growth patterns were similar; a 2-d exponential phase of considerable growth (2.5- and 3-log increase, respectively) followed by a steep decline and termination at 6 dpi (Fig. 3c). While under a starting pH of 4.5 the growth increased starting at 1dpi, the growth under pH conditions of 5.0 minimally decreased at d 1 before sharply increasing (Fig. 3c).

Under starting pH conditions of 4.5 and 5.0, the pH of the culture declined after d 1, dropping to a pH of 3.9 on d 3 and remained relatively stable through 6 dpi (Fig. 3b). The decline in the pH of both cultures seems to correlate with the decline in growth as the CFU dropped somewhat at 3 dpi from a high at 2 dpi and precipitously by 4 dpi with loss of viability by 6 dpi (Fig. S2e,f).

#### L. crispatus JV-V01 grew for 30 d in MSVF only at pH 5.0

**Fig. 4:**
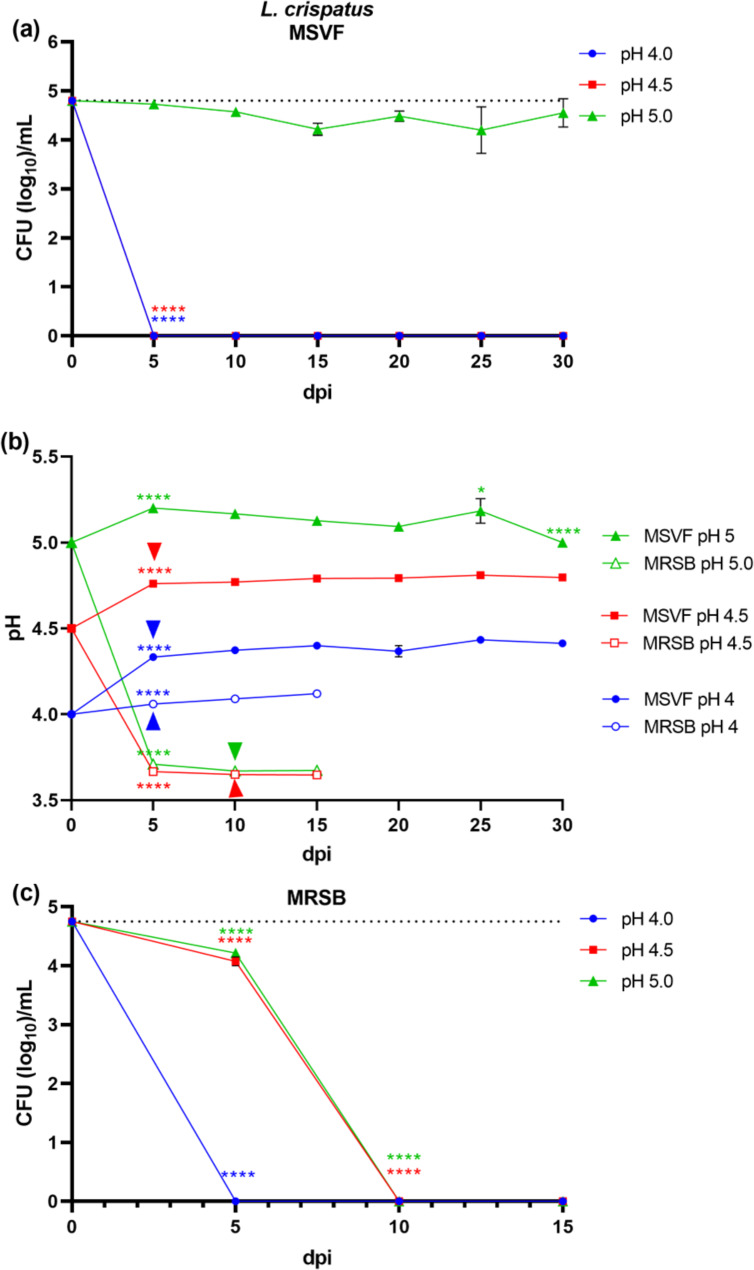
Growth patterns and changes in pH of *L. crispatus* JV-V01 grown in MSVF and MRSB. *L. crispatus* was grown overnight in MRSB at pH 6.3 and processed as described in Fig. 1. **(a)** Viability of *L. crispatus* grown in MSVF for 30 d at starting pH of 4.0, 4.5, and 5.0; **(b)** pH of MSVF and MRSB at each time point throughout the growth cycle; **(c)** viability of *L. crispatus* grown in MRSB for 30 d at starting pH of 4.0, 4.5, and 5.0. Each symbol represents the mean of three independent experiments ± SOM. Dotted lines (a, c) indicate starting CFU/mL. Arrowheads (b) indicate loss of viability. Two-way ANOVA with Tukey’s multiple comparisons posttest was done to determine significant differences between time points across the growth curves. *, *P* < 0.05; ***, *P* < 0.001; ****, *P* < 0.0001

Similar to *L. gasseri* and at a starting pH of 4.0, we recovered no viable colonies of *L. crispatus* JV-V01 at 5 dpi (Fig. 4a). However, unlike *L. gasseri* and *L. jensenii* in MSVF, the growth of *L. crispatus* was unique in that the growth under pH conditions of 4.5 was not supported (Fig. 4a). The loss of viable colonies at either starting pH (4.0 or 4.5) possibly occurred earlier than the 5-dpi time point.

Under starting pH conditions of 5.0, viable colonies were maintained throughout the growth cycle with very limited variation (less than 0.5 log) over 15 dpi, resembling an extended lag phase (Fig. 4b). At pH conditions of 5.0, the pH of the culture remained essentially the same throughout the growth cycle (Fig. 4b). As with the other species, there was little correlation between pH and CFU (Fig. S1h).

#### L. crispatus JV-V01 did not survive for 30 d in MRSB at any pH

The growth pattern under starting pH conditions of 4.0 was like that in MSVF; no viable colonies at 5 dpi (Fig. 4c). At starting pH conditions of 4.5 and 5.0, the growth patterns were identical; a 0.5 log reduction in growth on 5 dpi followed by no recoverable CFU on d 10 (Fig. 4c).

At 5 dpi, under starting pH conditions of 4.5 and 5.0, the pH of the culture had dropped to 3.8 and remained the same throughout the 15-d period (Fig. 4b). This reduction in pH appears to correlate with the reduction in growth at 5 dpi and loss of viability at 10 dpi (Fig. S2g, h).

#### Glycogen is essential for the viability of G. vaginalis JCP8151A, L. jensenii 62G, L. gasseri 63 AM, and L. crispatus JV-V01 under different starting pH conditions

Glycogen, abundantly found in vaginal fluid, is an essential carbon source for the growth and maintenance of vaginal microorganisms including lactobacilli and *G. vaginalis* [25, 26]. Similarly, glycogen, together with glucose, is included in MSVF as an essential carbon source [38]. Therefore, it is possible that the above observed variation in growth and/or viability of the lactobacilli as well as *G. vaginalis* under different starting pH conditions is due the influence of the starting pH conditions on glycogen utilization by the tested strains. To test this possibility, we grew all four strains (individually) in MSVF with a reduced glycogen concentration (5 g/L, MSVF_5Gly) or MSVF without glycogen (MSVF_0Gly) under all three pH conditions.

**Fig. 5:**
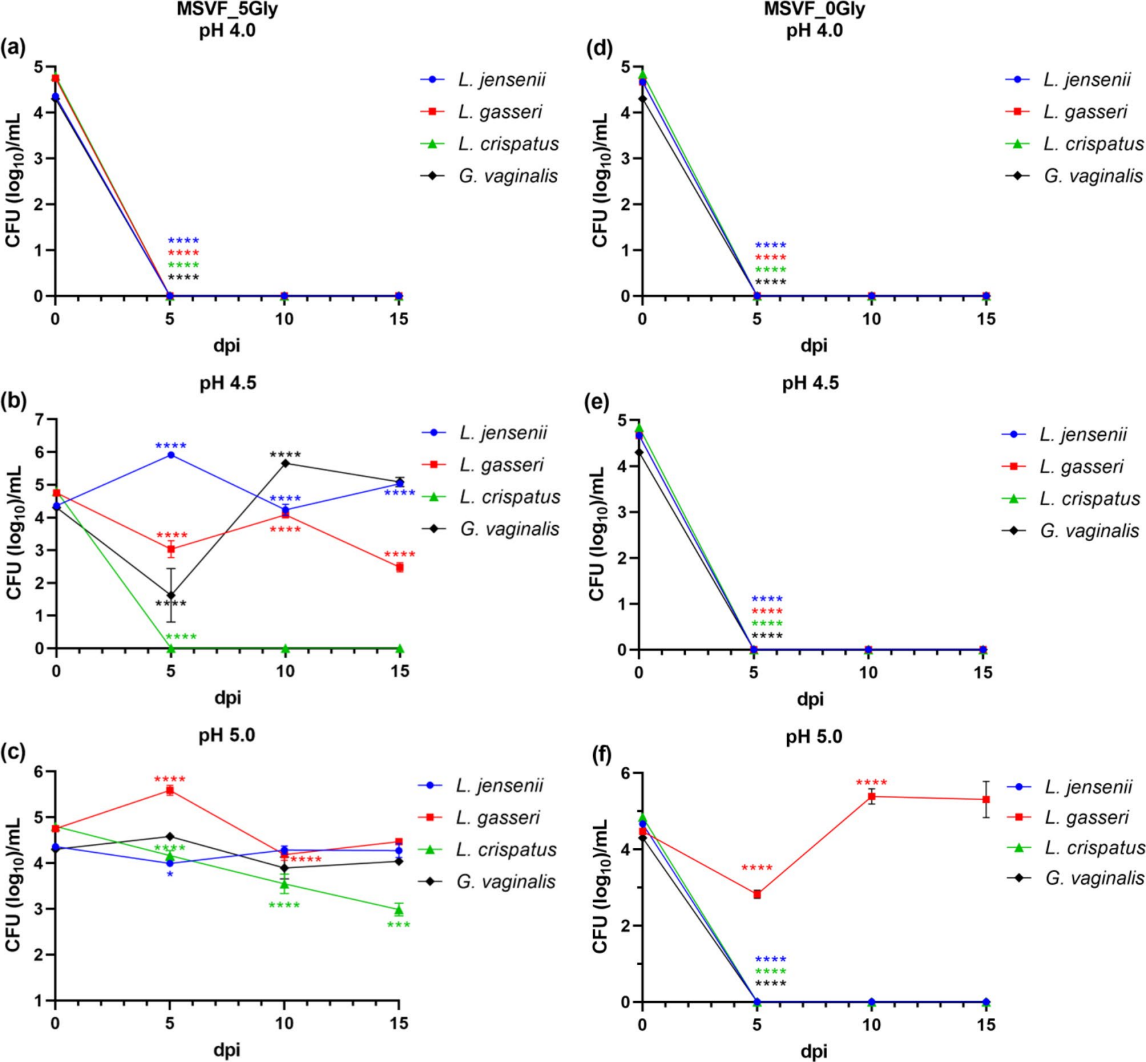
All strains require glycogen for growth except *L. gasseri* 63 AM, which uses glucose at pH 5.0. Strains were grown for 15 d in standard MSVF, which contains 10 g/L of glycogen and 10 g/L of glucose, MSVF with half the level of glycogen (MSVF_5Gly) at **(a)** pH 4.0, **(b)** pH 4.5, and **(c)** pH 5.0, and MSVF with no glycogen (MSVF_0Gly) at **(d)** pH 4.0, **(e)** pH 4.5, and **(f)** pH 5.0 Samples were taken at 5-d intervals and the CFU/mL were determined. Each symbol represents the mean of three independent experiments ± SOM. Two-way ANOVA with Tukey’s multiple comparisons posttest was done to determine significant differences between time points across the growth curves. *, *P* < 0.05; ***, *P* < 0.001; ****, *P* < 0.0001

Similar to the growth of *G. vaginalis* in standard MSVF (Fig. 1a), under starting pH conditions of 4.0, *G. vaginalis* viability was lost at the first time point (5 dpi), in MSVF_5Gly (Fig. 5a). In MSVF_5Gly pH 4.5, the pattern of growth was similar to that in the standard MSVF (Fig. S3a), with the exception of an extended recovery period (10 d as opposed to a 2-d recovery, respectively) (Fig. 5b). In MSVF_5Gly pH 5.0, there was no major difference in the growth throughout the 15-d growth period (Fig. 5c, S3a). When we omitted glycogen from the MSVF and despite the presence of glucose, we failed to recover viable CFU under starting pH conditions of 4.0, 4.5, or 5.0 beyond 5 dpi (Fig. 5d-f). At all time points, variations in the level of glycogen did not impact the pH of the cultures under any of the starting pH conditions where growth occurred (Fig. S4a, b). These results suggest that in MSVF glycogen is critical for *G. vaginalis* viability and that *G. vaginalis* is adapted to utilize glycogen but not glucose as a carbon source.

Under starting pH conditions of 4.0 and similar to *G. vaginalis*, we failed to detect viable *L. jensenii* at 5 dpi in either MSVF_5Gly or MSVF_0Gly (Fig. 5a, d). In MSVF_5Gly at pH 4.5, *L. jensenii* growth peaked at 5 dpi (Fig. 5b). After this peak, the growth leveled off with only a 0.5 log difference between 10 and 15 dpi (Fig. 5b, S3b). The growth under starting pH condition of 5.0 in MSVF_5Gly is almost identical at each of the different time points (Fig. 5c, S3b). In the absence of glycogen, we recovered no viable *L. jensenii* CFU at starting pH conditions of either 4.5 or 5.0 (Fig. 5e, f). Like *G. vaginalis*, there was no impact on the pH of the cultures (Fig. S4a,b).

Under starting pH conditions of either 4.0 or 4.5 and in MSVF_5Gly, we recovered no viable *L. crispatus* CFU (Fig. 5a,b). Under starting pH conditions of 5.0, the viability pattern in the presence of MSVF_5Gly is different from that in standard MSVF (Fig. S3c). While the growth pattern is constant in MSVF, in MSVF_5Gly the viability gradually declined from ~ 10^5^ CFU/mL at the initial inoculation point to 10^2^ at 15 dpi (Fig. 5c, S3d). It is possible, that at a concentration of 5 g/L, glycogen only partially supports the viability of *L. crispatus* and that if the growth curve were extended over time, the CFU would reduce further. Similar to *G. vaginalis* and *L. jensenii*, *L. crispatus* viability was lost in MSVF_0gly (Fig. 5d-f). As seen with MSVF, no drop in pH occurred in MSVF_5Gly (Fig. S4b).

Like other tested strains and under starting pH conditions of 4.0, no viable *L. gasseri* was recovered in MSVF_5Gly or MSVF_0Gly at 5 dpi (Fig. 5a, d). In MSVF_5Gly, we recovered viable *L. gasseri* colonies under starting pH conditions of 4.5 and 5.0 (Fig. 5b, c). However, compared with MSVF, MSVF_5Gly was less efficient in sustaining *L. gasseri* viability at pH 4.5 (Fig. S3d). Over the 15-d period, the growth of *L. gasseri* at pH 4.5 in MSVF_5Gly decreased by two logs compared to no reduction in MSVF (Fig. S3d). In MSVF_5Gly at pH 5.0, the growth was similar to that in MSVF, except that from 0 to 15 dpi there was a 0.5 log reduction in growth and not a 0.5 log increase like in MSVF (Fig. S3f). Surprisingly, and in contrast to *G. vaginalis* and other tested lactobacilli, *L. gasseri* grew even in MSVF_0Gly, but only at a starting pH of 5.0 (Fig. 5f). After an initial recovery period at 5 dpi, the growth increased by two logs at 10 dpi and stayed the same at 15 dpi (Fig. 5f). This suggests that during the growth in MSVF, and possibly in vivo, *L. gasseri* contains a unique, pH-sensitive glucose utilization mechanism; functioning under pH conditions of 5.0, but not 4.5 and lower. In contrast to its glucose usage in MRSB, glucose metabolism in MSVF_0Gly did not affect the pH (Fig. S4c).

#### L. gasseri 63 AM utilizes glucose as an alternative carbon source in the absence of glycogen

**Fig. 6:**
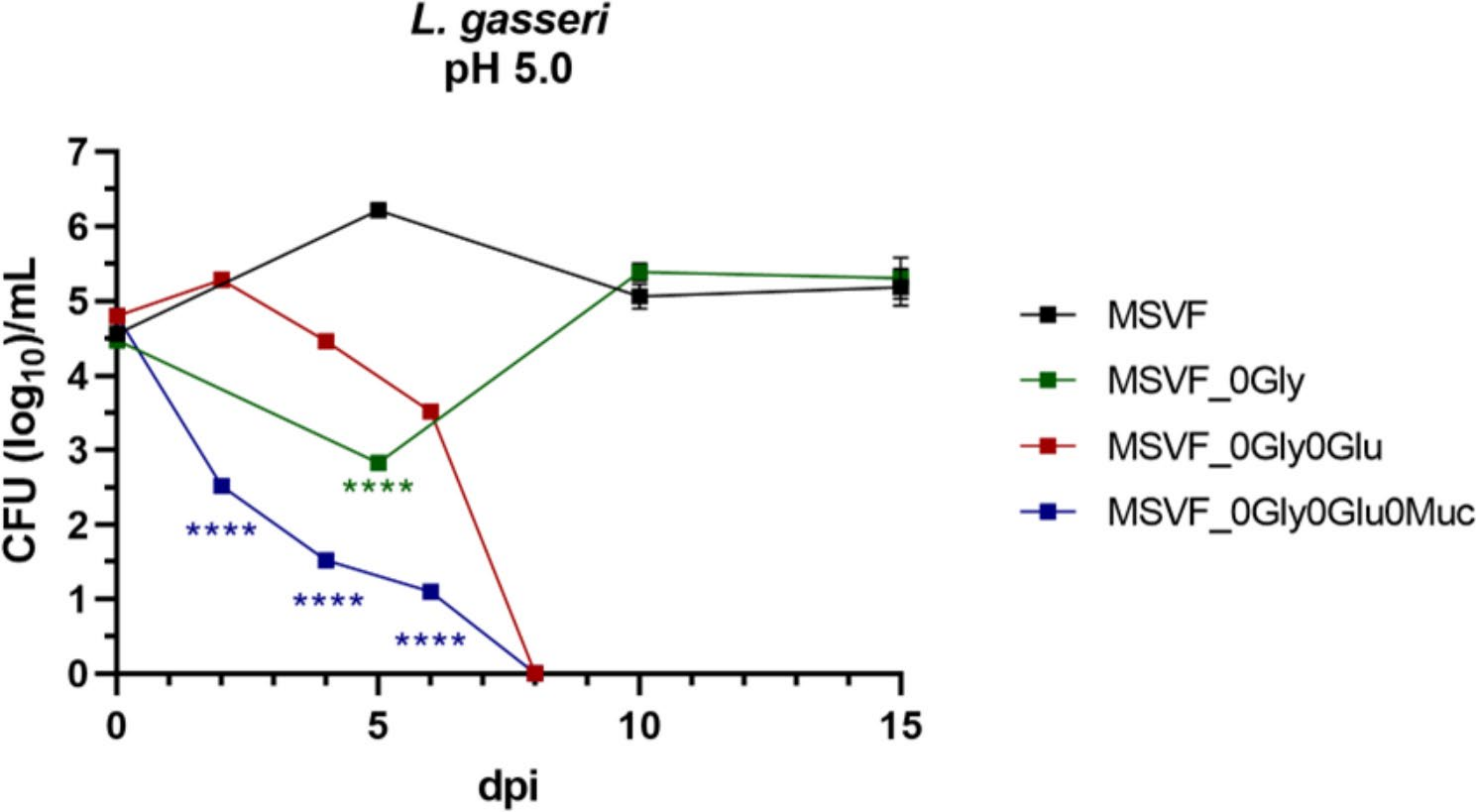
* L. gasseri* 63 AM utilizes alternative carbon sources within MSVF pH 5.0. Growth of *L. gasseri* in standard MSVF was compared to its growth in MSVF_0Gly, MSVF without glycogen or glucose (MSVF_0Gly0Glu), and without glycogen, glucose, or mucin (MSVF_0Gly0Glu0Muc) over 15 d, or until viability was lost. Each symbol represents the mean of three independent experiments ± SOM. Two-way ANOVA with Šídák’s multiple comparisons posttest was done to determine significant differences between time points across the growth curves; ****, *P* < 0.0001

*L. gasseri* 63 AM not only survived in MSVF_0Gly, but the CFU were similar to those recovered in MSVF at 10 and 15 dpi **(**Fig. S3d**)**. Therefore, it may be, that in the absence of glycogen, *L. gasseri* is the only tested strain that can utilize glucose as a carbon source, but only at a starting pH condition of 5.0 (Fig. 5f). To investigate this further, we formulated MSVF containing neither glycogen nor glucose (MSVF_0Gly0Glu). In this medium and under starting pH conditions of 5.0, *L. gasseri* viability increased by 0.5 logs at 2 dpi followed by a decrease in growth at 4 and 6 dpi and loss of viability at 8 dpi **(**Fig. 6**)**. Carrying this premise further, we removed mucin, a third carbon source, from MSVF_0Gly0Glu (MSVF_0Gly0glu0Muc) and examined the growth of *L. gasseri*. Unlike the increase at 2 dpi seen in MSVF_0Gly0Glu, in MSVF_0Gly0Glu0Muc there was no increase in growth, instead exponential decrease characteristic of the death phase occurred from 0 to 8 dpi **(**Fig. 6**)**. Together, these results suggest that at a pH of 5.0 *L. gasseri* can use alternative carbon sources to maintain viability in times when the preferred nutrient (glycogen) is limited.

#### Both L. jensenii 62G and G. vaginalis JCP8151A are metabolically active during the prolonged stationary phase

**Fig. 7:**
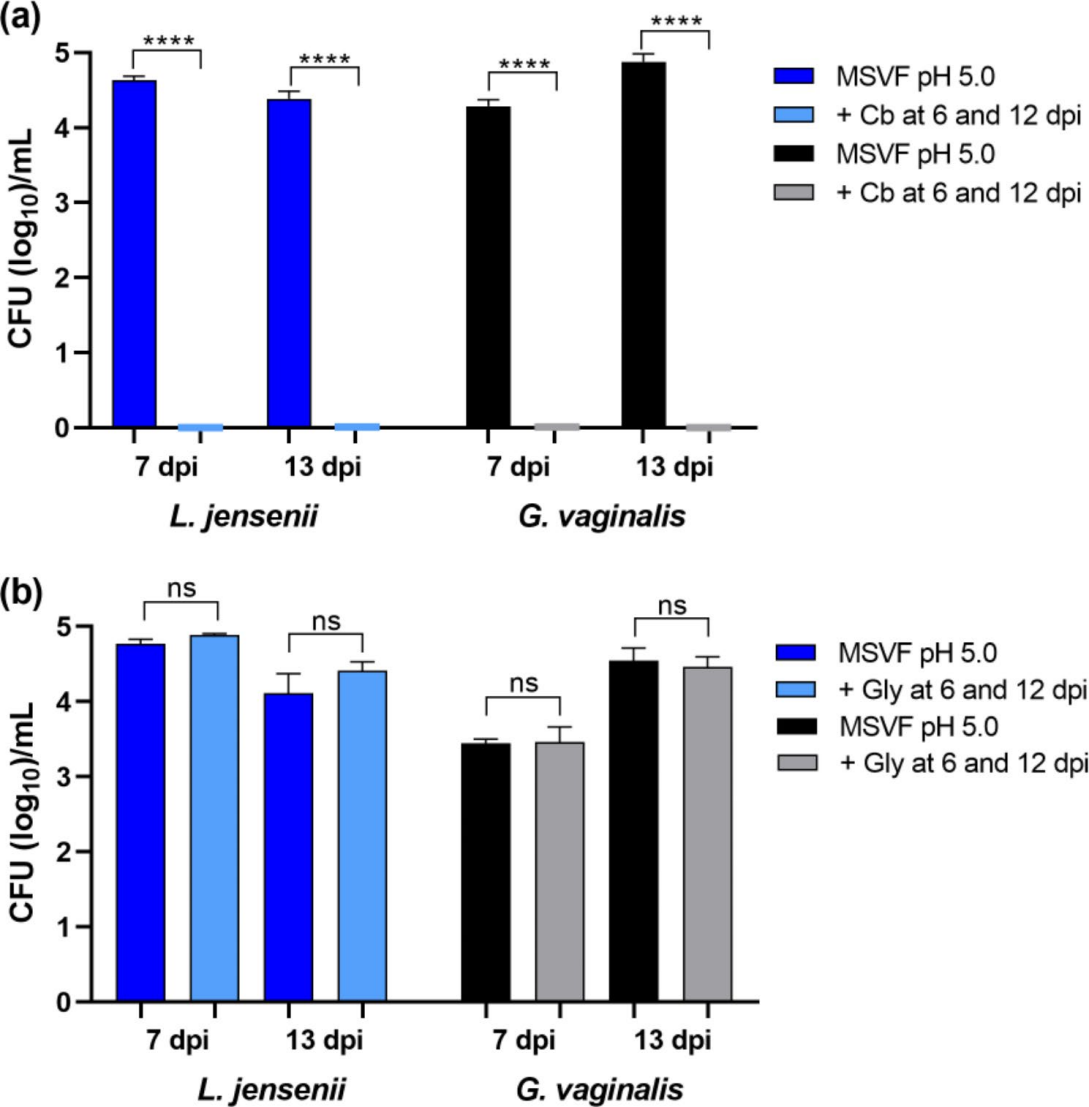
* L. jensenii* 62G and *G. vaginalis* JCP8151A are metabolically active during the long-term stationary phase. **(a)** Strains were grown in MSVF at pH 5.0 for 6 and 12 dpi and carbenicillin sufficient to inhibit growth (64 µg/mL for *L. jensenii* or 1 µg/mL for *G. vaginalis*) was added. Incubation was continued for an additional 24 h and samples were taken at 7 and 13 dpi and the CFU/mL were determined. **(b)** Addition of exogenous glycogen to MSVF pH 5.0 at intervals during the long-term stationary phase did not affect growth of *L. jensenii* or *G. vaginalis*. A concentrated solution of glycogen in water to a total of 2.5 mg/mL was added to cultures of the strains in MSVF at pH 5.0 at 6 or 12 dpi. Incubation was continued for an additional 24 h and samples were taken at 7 and 13 dpi and the CFU/mL were determined. For both panels: each symbol represents the mean of three independent experiments ± SOM. Two-tailed unpaired *t-*tests were used to determine significant differences between treatments; ns, no significance; ****, *P* < 0.0001

Regardless of starting pH, when they grew in MSVF, each strain showed sustained survival for 30 dpi in the LTSP that is considerably longer that the usual stationary phase of 16–24 h stationary phase of bacterial growth in nutrient rich medium [40, 42]. Following peak growth between 2 and 4 dpi, growth of *G. vaginalis* JCP8151A and *L. jensenii* 62G was characterized by a slow and fluctuating decrease in CFU (Figs. 1a and 2a). One possible explanation for these findings is that during the LTSP, essential nutrients are depleted forcing the bacteria to enter a dormant or a starvation mode, during which they are metabolically inactive [41]. The loss of metabolic activity can be demonstrated through the non-responsiveness of the bacteria to antibiotics that require metabolically active cells for their function as shown previously with *Bacillus subtilis* [41]. *B. subtilis* in a starvation buffer lacking carbon source, showed considerably increased tolerance to the aminopenicillin ampicillin, whereas cells in the exponential phase were efficiently killed by ampicillin [41]. Therefore, to assess the metabolic state of cells at different time points during the prolonged stationary phase, we examined the sensitivity *L. jensenii* 62G and *G. vaginalis* JCP8151A grown in MSVF under starting pH of 5.0 to the carboxypenicillin carbenicillin by adding carbenicillin, at concentrations that we had previously determined to be inhibitory (data not shown), at 6 or 12 dpi and determined the CFU 24 h later. At both time points, the addition of carbenicillin reduced the CFU from greater than 4 logs to 0, indicating that at both time points *L. jensenii* and *G. vaginalis* cells are metabolically active (Fig. 7a).

We also examined the possibility that the prolonged stationary phase is due to the depletion of an essential nutrient such as glycogen by adding glycogen at a concentration of 2.5 mg/µL to cultures of *L. jensenii* 62G and *G. vaginalis* JCP8151A grown in MSVF with starting pH of 5.0 at 6 or 12 dpi and analyzed the viability 24 h after the addition of glycogen. Compared to its continued growth in MSVF, the addition of glycogen produced no increase in CFU with *L. jensenii* or *G. vaginalis* at either time point, suggesting that the observed phenomenon is not induced by nutrient depletion (Fig. 7b).

## Discussion

While water is the main component of vaginal fluid, the many other constituents include compounds that support the growth of the vaginal flora including glycogen, glucose, and mucin [34]. In women of child-bearing age, and when *Lactobacillus* spp. dominate the vaginal microbiota (eubiosis), lactic acid produced by the vaginal lactobacilli as an end product of carbohydrate metabolism maintains the low pH of the vaginal fluid (generally < 4.5) [1–4, 6]. The dysbiotic condition BV, which is classically associated with a rise in vaginal pH above 4.5, occurs when the environment within the vagina changes to favor the overgrowth of facultative (*G. vaginalis*) and strict anaerobes [5, 9–11]. The main purpose of this study was to investigate the viability of selected species of vaginal lactobacilli and *G. vaginalis* in MSVF, a medium that mimics vaginal fluid and includes glycogen, glucose, and mucin [38], and the effect of physiologic pH changes in MSVF on both viability and glycogen usage.

Originally, MSVF was used to examine the growth of *L. gasseri, Lacticaseibacillus paracasei, Ligilactobacillus salivarius, L. johnsonii*, and *L. acidophilus*, but not *L. crispatus* or *L. jensenii*, or *G. vaginalis*; and that analysis was conducted under different experimental conditions – a 72 h growth period in MSVF at a pH of 4.2–4.3 [38]. Our findings clearly demonstrate that at specific physiological pH levels associated with eubiosis (pH 4.0, 4.5, and 5.0) [43], MSVF supported the growth of the three vaginal lactobacilli, *L. jensenii* 62G, *L. gasseri* 63 AM, and *L. crispatus* JV-V01, and *G. vaginalis* JCP8151A over a prolonged period similar to the length of the menstrual cycle (30 d) (Figs. 1a, 2, 3 and 4a). While MSVF supported the growth of all four species for 30 d in this study, the viability of each species was dependent on pH: *L. crispatus* survived for 30 d only at pH 5.0; *L. gasseri* and *G. vaginalis* survived for 30 d at pH 4.5 and 5.0 but not at pH 4.0; and *L. jensenii* survived for 30 d at pH 4.0, 4.5, and 5.0 (Figs. 1a, 2, 3 and 4a). The previously unreported finding, that MSVF also supports growth of *G. vaginalis*, validates the suggested reflectiveness of this medium to the conditions within the vaginal fluid [38]. Furthermore, the growth of *G. vaginalis* at pH 4.5 and 5.0 corroborates the finding that *G. vaginalis* occurs among the members of the CSTs of the vaginal microbiome at pH 4.5 and higher [2]. Our results also confirm the suitability of MSVF to mimic in vivo-like conditions for analysis of the growth of different sets of vaginal microorganisms and the interplay among those microorganisms under the various pH conditions commonly observed in the vaginal environment during eubiosis as well as dysbiosis. This study represents the first comprehensive analysis of these three specific vaginal lactobacilli plus *G. vaginalis* in a medium that mimics vaginal fluid, such as MSVF, at pH levels found in the vagina.

The growth patterns of the three *Lactobacillus* spp. and *G. vaginalis* in MSVF compared to those in the laboratory media MRSB and NYCB, respectively, revealed that MSVF was much more supportive of all four species overall, and that pH affected the growth of each species in the two media (Figs. 1, 2, 3 and 4). When grown in MSVF, all three *Lactobacillus* spp. survived for 30 dpi at starting pH 5.0, all except *L. crispatus* at pH 4.5, but only *L. jensenii* remained viable at pH 4.0 (Figs. 2a, 3 and 4a). In contrast, when grown in MRSB none of the three species survived beyond 5 dpi at pH 4.0; and only *L. jensenii* was viable for 30 dpi in MRSB at pH 4.5 and 5.0 (Figs. 2c, 3 and 4c). The growth of *G. vaginalis* in MSVF compared to NCYB was similar to and different from the patterns of the lactobacilli: there was no survival in either medium at starting pH 4.0; growth in both media at pH 4.5 was characterized by a striking decline in CFU early in the growth cycle; and similar to *L.* gasseri in MRSB pH 5.0, a high peak in CFU at 5 dpi was followed by death of the culture by 8 dpi in NYCB pH 5.0 (Figs. 1a and c and 3c). Compared to their growth in MSVF versus MRSB or NYCB, there was considerable variation in the CFU of each species at the same time points, at times higher in MSVF, at times parallel, and often lower (Figs. 1, 2, 3 and 4).

The growth patterns of *L. jensenii* in MSVF at all pH levels was characteristic of the long-term stationary phase (LTSP) in which the lag, exponential, stationary, and death phases are followed by rising and falling numbers of CFU that remained around the level of the starting inoculum over the remaining growth cycle (Fig. 2a) [44–46]. In MSVF at pH 4.5 and 5, *G. vaginalis* also exhibited growth patterns leading to the LTSP as did *L. gasseri* in MSVF at pH 5.0 (Figs. 1a and 3a). However, a different growth pattern was displayed by *L. gasseri* in MSVF pH 4.5 and by *L. crispatus* in MSVF at pH 5.0; the bacteria appeared to remain in an extended lag phase with no appreciable rise (or fall) in CFU (Figs. 3a and 4a) [47]. Both the LTSP and the extended lag phase are survival mechanisms exhibited by bacteria under stress, whether nutritional, antibiotic-induced, or other stress [45, 47]. The ability of MSVF to maintain the long-term stationary phase and/or the extended lag phase also supports the use of the MSVF medium for future studies as the numbers of individual species within the vaginal microbiota remains relatively stable, but fluctuations occur over time reflecting nutrient- and pH-related conditions [2, 20, 48].

In contrast, in the defined laboratory medium MRSB, which lacks glycogen and mucin, none of the species survived beyond 5 dpi at pH 4.0; and at pH 4.5 and 5.0, only *L. jensenii* entered the LTSP observed in MSVF at the same pH levels (Figs. 1c, 2, 3 and 4c). In MRSB at pH 4.5 and 5.0, *L. gasseri* growth patterns were more like the classical pattern ending in death by 6 dpi while *L. crispatus* growth was not well-supported by MRSB, as the organisms had entered death phase by 5 dpi (Figs. 3c and 4c). Similarly, *G. vaginalis* grown in NYCB, which also lacks glycogen and mucin, did not survive at pH 4.0 and exhibited the classic growth curve at pH 5.0 with death by 8 dpi (Fig. 1c). As seen in MSVF at pH 4.5, in NYCB at pH 4.5, *G. vaginalis* also entered the LTSP by 10 dpi (Fig. 1b). Both *L. jensenii* (when grown in MRSB) and *G. vaginalis* (whether grown in MSVF or NYCB) exhibited a decline in CFU at 1 dpi at starting pH 4.5 (Figs. 1a and c and 2c). The drop in CFU is indicative of the “decline phase” that may precede the lag and exponential phases as organisms adapt to the stress of a new environment [40, 49]. As MRSB is standardized for the growth of lactobacilli at pH 6.3 and NCYB for the growth of *G. vaginalis* at pH 7.3, these declines are likely to represent adaptation to the abrupt change of pH from the overnight cultures to pH 4.5. Thus, pH as well as substrate availability produced marked differences in the growth of *L. jensenii*, *L. gasseri*, *L. crispatus*, and *G. vaginalis*.

With respect to the short period of viability of *L. gasseri* and *L. crispatus* in MRSB at all pH levels and *G. vaginalis* in NYCB at pH 5.0, it is possible that their utilization of nutrients within the media resulted in byproducts that negatively affected their ability to survive. We found an association between a drop in MRSB pH from either 4.5 to 3.8 or 5.0 to 3.7 and the loss of *L. gasseri* and *L. crispatus* viability, respectively; and a drop in NYCB pH from 4.5 or 5.0 to 4.4 and the loss of *G. vaginalis* (Fig. S2b,e-i). Using what they termed “vaginal defined medium” that was supplemented with peptone, Pybus & Onderdonk showed that *G. vaginalis* produced a detectable level of acetate when its growth reached levels of 10^7^ CFU/mL and that only when acetate was detectable was there an associated decrease in pH [50]. It has also been shown that *E. coli* produces acetate when levels of glucose are in excess [51]. At high levels, acetate acts as an uncoupler of the proton motor force, causing the cells to pump protons out of the cells in order to maintain membrane potential; this effort results in decrease in the pH of the culture to the detriment of the cells [52, 53]. It is possible that at pH 5.0 in NYCB, *G. vaginalis* metabolizes the glucose and/or the peptone in the medium to produce acetate causing the pH level to drop, thus eliminating their growth. Whether acetate production is the cause of the pH drop or not, the death of the *G. vaginalis* cultures suggests that pH 4.5 is the lower limit for its survival, which may explain why it is not present in *L. crispatus-*dominated CST I with a pH range of 3.7–4.3 in vivo [2]. Like NYCB, MRSB also contains glucose and peptone suggesting acetate formation may occur during the growth of *L. gasseri* and *L. crispatus*.

Neither MRSB nor NYCB contain glycogen as a carbon source although the vaginal lactobacilli and *G. vaginalis* have been shown to rely heavily on this nutrient, which is the most abundant of the host-derived nutrients present in vaginal fluid [27, 30, 31]. Thus, the inability of the vaginal bacteria to grow beyond 10 dpi in the laboratory media is at least partly due to this lack of an essential nutrient or to the inability of the species to switch to a different substrate (i.e., glucose, which is present in all three media) for energy. Furthermore, our results show that, not only is glycogen essential for the growth of *L. jensenii* 62G, *L. gasseri* 63 AM, and *L. crispatus* JV-V01 and *G. vaginalis* JCP8151A, but they also suggest that the pH of the medium influences their viability by affecting their ability to metabolize glycogen (Fig. 5, S3).

Glycogen levels within vaginal cells increase at puberty under the influence of estrogen, leading to higher levels of glycogen in the vaginal fluid [25–27]. As the amount of glycogen increases, the numbers of *Lactobacillus* spp. within the vaginal microbiome increase and the low pH associated with a healthy adult vaginal microbiome develops [6, 8, 26, 29, 54]. Glycogen, a large branched D-glucose polymer, is digested by glycoside hydrolases (GH) that are divided into 128 families based on their substrate specificity, stereochemistry of the catalyzed reaction, site of action, and sequence or structure (Carbohydrate Active Enzyme database; http://www.cazy.org/; accessed 20 Feb 2023) [55–57]. The human glycogen-digesting enzyme α-amylase, a member of GH Family 13, has long been known to be present within human vaginal fluid [58, 59]. This enzyme, which cleaves ⍺-1,4 glycosidic bonds, has been presumed for decades to be the source of the glycogen metabolic products maltose, maltotriose, and glucose utilized by the lactobacilli and other vaginal bacteria [29, 60]. In 2005, Oh et al. characterized a maltogenic amylase from *L. gasseri* and in 2019, van der Veer et al. described the presence of a type I pullulanase gene (PulA) within the *L. crispatus* genome [61, 62]. Both enzymes cleave α-1,6 bonds of glycogen and are members of GH Family 13, suggesting that some lactobacilli may be capable of glycogen metabolism on their own [61, 62]. Another recent study has provided evidence that strains of *L. crispatus*, *L. jensenii*, and *L. gasseri*, as well as *G. vaginalis*, all carry multiple (at least three) genes for GHs that would allow for their direct utilization of glycogen [63]. Together, these studies [61–63] contradict the previous theory that glycogen can only be used by vaginal bacteria when it has been broken down by human α-amylase [29, 58–60]. Further confirmation that vaginal bacteria carry functional glycogen-digesting enzymes has come from characterization of amylopullanases from *L. crispatus, L. iners* and *G. vaginalis* [64–66]. Each of the enzymes encoded by the different species we tested utilize glycogen (among others) as a substrate, cleaving it to maltose, maltotriose, malto-oligosaccharides, trehalose, limit dextrin and/or glucose, depending on the specific enzyme (BRENDA database, https://www.brenda-enzymes.org/index.php, accessed 20 Feb 2023) [55, 56, 67, 68]. Additionally, several of these enzymes can cleave the glycogen breakdown products such as maltose and maltotriose to glucose [68]. Additionally, each species carries enzymes that cleave α-1,4-glycosidic linkages, α-1,6-glycosidic linkages, or both, providing a number of mechanisms by which the bacteria can utilize glycogen for energy [56, 63, 68]. Thus, it is more likely that glycogen utilization by the vaginal microbiota occurs through a combination of both bacterial and host enzymes.

The presence and types of glycogen utilization proteins varies from species to species and strain to strain within *L. jensenii, L. gasseri, L. crispatus*, and *G. vaginalis* [61–63], which may affect the ability of these organisms to utilize glycogen within different growth media and under different pH conditions. As the genomes of the strains used in this study have been published and translated proteins are available in both the NCBI non-redundant protein database (https://www.ncbi.nlm.nih.gov/) and UniProt (https://www.uniprot.org/), we performed an *in silico* search for glycogen utilization proteins described above within our tested strains. (see Additional File 1 for methods and references). Proteins with ≥ 40% identity or ≥ 50% similarity and ≥ 75% coverage were considered potential matches and are shown in Table S1. Depending on the reference protein used for the search (type I pullulanase (PulA), isoamylase, or neopullanase), homologous proteins for type I pullulanases were found in *L. jensenii* 62G (aka DSM 20,557), *L. gasseri* 63 AM, and *L. crispatus* JV-V01 and *G. vaginalis* JCP8151A (Table S1). Deletions within the *N* terminus region of PulA have been shown to occur in many clinical isolates of *L. crispatus*, rendered those strains incapable of utilizing glycogen [62]. Therefore, we determined that the *N-*terminal region of *L. crispatus* JV-V01 type I pullulanase protein PulA (EEJ69081.1) was intact, suggesting that the enzyme is functional (Fig. S6) [62]. Matches for α-glucosidase, α-glycosidase, and intracellular maltogenic amylase were found only in *L. jensenii* 62G, *L. gasseri* 63 AM, and *L. crispatus* JV-V01, while matches for glycogen debranching enzyme and α-amylase were found only in *G. vaginalis* JCP8151A (Table S1). Despite the presence of a type II pullulanase in *L. iners* L1 (MK770623.1) and *L. crispatus* [64], we retrieved no matches to type II pullulanases from any of our tested strains (all matches were type I pullulanases) (Table S1). More than one enzyme was present in each strain, suggesting that these enzymes enabled these strains to utilize glycogen. Whether other strains of these species would behave in the same manner as the four specific strains we tested requires further investigation.

Each CST of the vaginal microbiota is associated with a specific pH range of the vaginal fluid, with the pH of CST I ranging from 3.7 to 4.3, that of CST II ranging from 4.3 to 5.7, CST III from pH 3.8 to 5.0, CST IV containing *G. vaginalis* ranging from pH 4.7 to 5.9, and CST V from 4.3 to 5.1 [19, 43]. Woolston et al. characterized amylopullanases (PulA) from *L. crispatus* (CST I), *L. iners* (CST III), and *G. vaginalis* (CST IV) and found that the enzymes were most active at pH 5.8 for the lactobacilli, which is much higher than the pH range established for CST I and CST III, and pH 6.0 (range 5.5–6.8) for *G. vaginalis* [19, 43, 65]. Thus, our results suggest that, at least in our system, the GH enzymes of the lactobacilli and *G. vaginalis* vary in their pH sensitivity, with *L. jensenii* 62G enzymes functional at all three pH levels, *L. crispatus* JV-V01 enzymes sensitive to pH 4.0 and 4.5, and *L. gasseri* 63 AM and *G. vaginalis* JCP8151A enzymes sensitive to pH 4.0 only (Figs. 1, 2, 3 and 4). It is possible that our conditions affected the pH tolerance of each strain. In addition, the pH sensitivity/resistance may change in a mixed species culture, which we plan to test next.

Our results showed that all three lactobacilli and *G. vaginalis* metabolized glycogen as an essential source of carbohydrate, as in the absence of glycogen there was reduced or no viability, except for *L. gasseri* in MVSF_0Gly at pH 5.0 (Fig. 5d-e). Furthermore, when we retained glycogen but eliminated glucose from MSVF (MSVF_0Glu), *L. jensenii* as well as *G. vaginalis* maintained viability (Fig. S5). This is likely due to the presence of multiple putative and/or characterized GHs that potentially allow for glycogen to be hydrolyzed at both ⍺-1,4- and ⍺-1,6- glycosidic bonds and that potentially function at different pH ranges covering the range of physiologic pH found in the vaginal fluid [61–66, 68–70].

Several studies suggested that due to the ability to maintain low pH conditions, *L. crispatus* significantly contributes to healthy vaginal conditions [2, 9, 71, 72]. During their analysis of vaginal bacterial communities of asymptomatic women from different ethnic groups, Ravel et al. showed that *L. crispatus* dominated CST I, which had the lowest pH median (4 ± 0.3) of the five CSTs [2, 9, 71, 72]. This raises a question regarding the mechanism through which these lactobacilli dominate their CSTs. Woolston et al. suggested that those strains may depend on cross feeding of oligosaccharides produced by glycogen degrading enzymes from either the host or other vaginal microbiota [65]. Thus, without this cross-feeding, *L. crispatus* growth is not as robust and is unable to continue at pH 4.0 or 4.5.

It is interesting that we did not detect this phenomenon when we grew *L. jensenii* in MRSB. Like its viability pattern in MSVF, *L. jensenii* maintained viability under pH conditions of 4.5 and 5.0 throughout the 30-d growth period with little change in the pH of the cultures (Fig. 2). Only at pH 5.0 and between 28 and 30 dpi was there a drop in pH from 5.0 to 4.6 which was accompanied by a one-log drop in CFU (Fig. S2d). This suggests that even when they are grown in MRSB, strains of lactobacilli vary both in their ability to metabolize the carbohydrate source and the level of acid they produce from their metabolism. In our study, the metabolic activity in MRSB or NYCB produced sufficient acidity to kill the bacteria early in the extended growth cycle; while in MSVF, the metabolism allowed the bacteria to maintain viability throughout the 30-d period.

Glycogen is not taken up directly by the lactobacilli or *G. vaginalis*. When placed in a new environment, intracellular GHs digest stored glycogen to maintain survival until the bacteria adapt to their new surroundings and can use extracellular carbon sources [47]. For organisms like the lactobacilli and *G. vaginalis* that require extracellular glycogen, several of the GH enzymes, such as maltogenic amylases, amylopullanases, and pullanases, are secreted into the surrounding environment where digestion of glycogen takes place [61–66, 69]. The larger breakdown products of glycogen digestion (maltose, maltotriose, maltotetraose, malto-oligosaccharides) and extracellular glucose are taken up by two distinct types of transport systems. Products of glycogen digestion are transported into lactobacilli through the four component ATP-binding cassette transport system MalEFG/MsmK [73, 74] while *G. vaginalis* has four separate transport systems for these products: the MusEFGK2I operon (maltose/maltotriose), the MalXFGK transporter (maltose, maltotriose, malto-oligosaccharides and maltodextrins), RafEFGK (α-1,6 linked glucosides and galactosides), and TMSP 322 (trehalose/maltose) [75]. Similar to *E. coli* and other bacteria, glucose can be transported into *L. jensenii*, *L. gasseri* and *L. crispatus* by the phosphoenolpyruvate: carbohydrate phosphotransferase system [76, 77] and into *G. vaginalis* by a putative glucose/galactose porter [75]. As with the glycogen utilization proteins, i*n silico* analysis revealed presence of transportation systems for glycogen breakdown products in our tested strains (Table S2). Matches for the MalEFG/MsmK transport system were found in *L. jensenii* 62G, *L. gasseri* 63 AM, and *L. crispatus* JV-V01 but not *G. vaginalis* JCP8151A except for a potential Malk/MsmK protein with 50% identity (Table S2). Similarly, matches were found in the three *Lactobacillus* stains for the accessory proteins MalP and PgmB, while MalH was found in *L. jensenii* 62G and *L. crispatus* JV-V01 but not *L. gasseri* as previously reported (Table S2) [73]. Proteins with 72–100% identity to the RafEFGK, MusEFGK2I and MalXFGK transport systems were found in *G. vaginalis* JCP8151A but not in the three lactobacilli we tested (Table S2).

Function of type II pullulanase enzymes and debranching enzymes from *L. crispatus* and *L. acidophilus*, respectively, has been shown to be repressed when the cells are grown on glucose but not when maltose or maltotriose is present [64, 78]. This does not seem to be the case for *L. jensenii and G. vaginalis*. The strains grew to higher CFU at 5 dpi and 10 dpi when both glucose and glycogen were present than when there was no glucose in the medium (MSVF_0Glu) (Fig. S5). The lesser growth may indicate internal glycogen usage until sufficient GH enzymes have been secreted to provide glycogen breakdown products for uptake. A puzzling finding in this study was our failure to detect viable *L. jensenii*, *L. crispatus*, or *G. vaginalis* when glycogen was removed from MSVF (MSVF_0Gly) despite the presence of glucose (Fig. 5d-f). Yet, both *L. jensenii* and *G. vaginalis* grew well in MRSB and NYCB, which also lack glycogen but contain glucose, at pH 4.5 and 5.0 (Figs. 1c and 2c), which suggests the presence of competent glucose uptake mechanisms. These are also rich media that contain numerous additional carbon sources, such as yeast extract and proteose [30, 31], that may allow their survival. Interestingly, in our system, *L. crispatus* did not thrive at pH 5.0 in MSVF (extended lag phase) or MSVF_5Gly (death phase), in which both glycogen and glucose are present nor in MRSB (rapid death) despite the richness of the medium (Figs. 3 and 5, S3c). Repression of GHs by glucose in MSVF may explain the flat growth curve [64], while the death phase in MSVF_5 and MRSB may indicate a requirement for a specific level of glycogen. Yet *L. crispatus* dominates CST I which exhibits the lowest pH (3.7–4.3) of any of the five vaginal CSTs, making a strong case for cross-feeding of glycogen breakdown products to it in the vaginal milieu [65]. It is likely that upon the initial growth of the *L. jensenii*, *L. gasseri*, and *G. vaginalis* in MSVF, a mixture of glucose and maltose, maltotriose, and other glycogen breakdown products would be present in the medium. Thus, it is possible that under these conditions and unlike MRSB and NYCB, lactobacilli and *G. vaginalis* suppress the glucose transport system and preferentially utilize the maltose transport system [69, 73, 74].

In the above paragraphs, we interpreted our results through the commonly recognized scenario in which the low pH of the healthy vaginal environment is maintained by the D- and L-lactic acid produced through anaerobic glycolysis of extracellular glycogen by different *Lactobacillus* strains [9, 79, 80]. However, the initial source of lactic acid within the vaginal fluid may be the host cells themselves. As early as the 1930s, it was shown that intracellular glycogen metabolism is responsible for the acid pH (5.0 or less) in the vaginal fluid [81, 82]. The anaerobic glycolysis of glycogen within vaginal epithelial cells leads to production of L-lactic acid which diffuses into the vaginal fluid [79, 80]. Vaginal pH of newborns is ~ 5.0 under the influence of maternal estrogen and shifts to neutral pH as the estrogen wanes [81, 82]. Similarly, the vaginal pH of post-menopausal women shifts to neutral with the loss of estrogen-stimulated glycogen deposition in the vaginal cells [81, 82]. Additionally, cervical cells lack glycogen, and the pH of the cervix is neutral despite the predominance of lactobacilli [80]. Further, it has been shown that even though lactobacilli may become numerous, and even preponderant, in the vaginal flora of prepubertal girls more than a year before their first menstrual cycle, the vaginal pH of some of these girls remained at pH 5.0 or above; only when glycogen was present within the vaginal epithelial cells did the pH drop below 5.0 [79]. As the amount of glycogen builds up in the vaginal epithelial cells, increasing amounts are diffused from the cells into the vaginal fluid where the lactobacilli and other bacteria can metabolize the glycogen into more lactic acid. Thus, the lactic acid in the vagina is contributed by both host cells and the vaginal microbiota [9, 79, 80].

## Conclusions

We demonstrated in this study that throughout the extended growth curve and when glycogen is present in sufficient amounts in MSVF, it is efficiently metabolized by *L. jensenii* under all starting pH conditions, by *L. gasseri* and *G. vaginalis* under starting pH conditions of 4.5 and 5.0, and by *L. crispatus* under starting pH condition of 5.0. Thus, based on these findings and those of other previous studies, we propose the following scenario for the sequential presence of the three lactobacilli and *G. vaginalis* within the vagina. A low pH (~ 4.0) may be produced in the vagina by the release of lactic acid from glycogen metabolism within the vaginal mucosal epithelial cells. Among the three *Lactobacillus* spp., and with its pH-tolerant glycogen-metabolizing enzymes, *L. jensenii* would metabolize the external glycogen and be the first species to increase its population within the vagina. External metabolization of the glycogen would release secondary metabolites that support the growth and colonization by other lactobacilli. Once the microbiome is more established, and glycogen breakdown products maltose, maltotriose, and malto-oligosaccharides are available, the GH enzymes may be able to use those substrates at a lower pH allowing *L. crispatus*, whose glycogen utilization functioned only at pH 5.0 in our system, to tolerate a lower pH and begin to dominate the microbiota. Besides the increase in the pH, other yet to be identified factor(s) within the vaginal fluid may enhance the growth of *G. vaginalis* at the expense of the lactobacilli resulting in the transition of the vaginal microbiota to produce BV. Despite its close resemblance to the vaginal fluid, MSVF likely lacks such a factor(s). Further experiments, including modifying our single species to a mixed species system, will be required to explore these possibilities.

## Materials and methods

### Bacterial strains and growth medium

*Gardnerella vaginalis* JCP8151A (BEI HM-1116) and *Lactobacillus crispatus* JV-V01 (BEI HM-103) obtained from the BEI Resources Repository (Manassas, VA, USA) and *Lactobacillus jensenii* 62G [Institute Pasteur 6917] (ATCC 25,258), *Lactobacillus gasseri* 63 AM (ATCC 33,323) obtained from the American Type Culture Collection (Manassas, VA, USA) were used in this study. The standard laboratory media New York City III broth (NYCB) (ATCC Medium 1685 formulation [https://www.atcc.org/]) and de Man, Rogosa, and Sharpe broth (MRSB) (Oxoid™, Thermo Scientific, Waltham, MA, USA) were used for *G. vaginalis* and the lactobacilli, respectively, as well as medium simulating vaginal fluid (MSVF) for all strains [30, 31, 38].

Frozen stocks of *G. vaginalis* or the different *Lactobacillus* spp. were inoculated into NYCB or MRSB, respectively, and incubated at 37℃ under 5% CO_2_ for 48 h. One-mL aliquots of the 48 h cultures were centrifuged and the pellets were washed three times in the medium to be used for the growth curve experiments (NYCB, MRSB, or MSVF) and resuspended in the same medium. The washed bacterial cells were then used to adjust fresh media to a starting OD_600_ of 0.02 to yield an initial inoculum of 10^4^ CFU/mL in 10 µL of culture for subsequent experiments.

### Extended growth curve experiments

Cultures were grown in MSVF, which closely mimic the vaginal fluid, for 30 dpi to reflect the 30-d cycle of conditions within the vagina; that is, the average menstrual cycle for women of child-bearing age, the group in which most BV occurs, is 28 d (21–35 d) [43, 83]. As pH critically affects the growth of vaginal microflora, we subjected the strains to pH conditions of 4.0, 4.5, and 5.0. The broth media were adjusted from their usual pH (6.3 for MRSB and 7.3 for NYCB) to pH levels of 4.0, 4.5 or 5.0 using acetic acid (NYCB and MRSB) or KOH (MSVF). One-mL aliquots of the media were pipetted into the wells of untreated 24-well microtiter plates (Costar, Corning, Durham, NC, USA) and inoculated with 10 µL of the prepared inocula described above; wells were inoculated in triplicate for each time point across the growth curve. Each plate was sealed with a Breathe-Easy membrane (Diversified Biotech, Dedham, MA, USA), to prevent desiccation over the extended incubation period (30 d). The plates were then incubated at 37℃ under 5% CO_2_ in a humid chamber for designated time periods. Samples were collected at 1-, 2-, or 5-d intervals depending on the species tested and the medium used to assess the viability and pH of the cultures. At specified time points, 60 µL of culture from individual wells were diluted tenfold and 10-µL aliquots were spotted in triplicate on chocolate agar (REMEL™, Thermo Scientific, Waltham, MA, USA) or MRS agar (Oxoid™) for *G. vaginalis* or *Lactobacillus* spp., respectively, to determine the number of CFU/mL. A 400-µL aliquot was used to determine the pH level of the respective cultures at each time point.

### Growth in different nutrient conditions

The MSVF medium contains 10 g/L of glycogen, 10 g/L of glucose and 0.25 g/L of mucin [38]. To assess the requirement for glycogen, MSVF was prepared without glycogen (MSVF_0Gly) or with 5 g/L of glycogen (MSVF_5Gly) and adjusted to pH 4.0, 4.5 or 5.0. Microtiter plates were prepared and inoculated individually with 10^4^ CFU/mL of each of the strains and incubated for up to 15 d. At 5-d intervals, the CFU/mL (viability) were determined, and the pH levels were measured.

Additional MSVF was prepared without glycogen or glucose (MSVF_0Gly0Glu), without glycogen, glucose, or mucin (MSVF_0Gly0Glu0Muc), and without glucose (MSVF_0Glu); these media were adjusted to pH 5.0. Microtiter plates were prepared and inoculated with 10^4^ CFU/mL of *L. gasseri* for MSVF_0Gly0Glu and MSVF_0Gly0Glu0Muc or 10^4^ CFU/mL of *L. jensenii* or *G. vaginalis* for MSVF_0Glu. The numbers of CFU/mL were determined MSVF_0Gly0Glu and MSVF_0Gly0Glu0Muc and at 5-d intervals over 15 d for MSVF_0Glu.

### Antibiotic or nutrient supplementation

To determine whether the bacteria in the prolonged stationary phase were metabolically active, the carboxypenicillin carbenicillin (Research Products International, Mount Prospect, IL, USA) was added to the media. *L. jensenii* or *G. vaginalis* was grown in MSVF at pH 5.0 for 6 d and 12 d, a predetermined concentration of carbenicillin sufficient to inhibit growth (64 µg/mL for *L. jensenii* or 1 µg/mL for *G. vaginalis*) was added to the cultures and incubation was continued for an additional 24 h (7 d or 13 d, respectively) at which time the numbers of CFU/mL were assessed.

To determine if glycogen limitation was affecting the growth of *L. jensenii* or *G. vaginalis*, the strains were grown in grown in MSVF at pH 5.0 for 6 d and 12 d and a concentrated solution of glycogen in water was added to the cultures to a total of 2.5 mg/mL. Incubation continued for an additional 24 h and the numbers of CFU/mL were assessed at 7 d or 13 d, respectively.

### Statistical analyses

GraphPad Prism version 9.4.0 (673) (GraphPad Software, San Diego, CA, USA) was used for all statistical analyses. Data represent the means ± SEM of three independent experiments for each group (*n* = 3). The CFU data were routinely log transformed prior to graphing and statistical analysis. Two-way ANOVA with Tukey’s multiple comparisons posttest or Šídák’s multiple comparisons posttest (as recommended by the program) was used to determine significance between time points in the growth curve analyses both for CFU and pH. One-way ANOVA with Dunnett’s multiple comparisons posttest was used to compare each treatment to fMSVF or appropriate control; one-way ANOVA with Tukey’s multiple comparisons posttest was used to compare differences among the fractions (all pairs of pairs). Two-tailed unpaired *t* tests were used to compare individual pairs. Statistical significance is shown as *, *p* < 0.05; **, *p* < 0.01; ***, *p* < 0.001; ****, *p* < 0.0001; ns, no significant difference.

## Electronic supplementary material

Below is the link to the electronic supplementary material.

Supplementary Material 1

Supplementary Material 2

Supplementary Material 3

Supplementary Material 4

## Data Availability

All data used and/or analyzed during the current study is available from the corresponding author on reasonable request.

## Declarations

### Authors’ information (optional)

None provided.

## Abbreviations

ATCC: American Type Culture Collection
BV: bacterial vaginosis
CFU: colony forming units
CST: community state type
dpi: days post inoculation
GH: glycoside hydrolases
*G. vaginalis*: *Gardnerella vaginalis*
*L. acidophilus*: *Lactobacillus acidophilus*
*L. crispatus*: *Lactobacillus crispatus*
*L. gasseri*: *Lactobacillus gasseri*
*L. iners*: *Lactobacillus iners*
*L. jensenii*: *Lactobacillus jensenii*
*L. johnsonii*: *Lactobacillus johnsonii*
LTSP: long-term stationary phase
MRSB: de Man, Rogosa, and Sharpe broth
MSVF: medium simulating vaginal fluid
MSVF_0Glu: MSVF without glucose
MSVF_0Gly: MSVF without glycogen
MSVF_0Gly0Glu: MSVF without glycogen or glucose
MSVF_0Gly0Glu0Muc: MSVF without glycogen, glucose, or mucin
MSVF_5Gly: MSVF containing 5 g/L of glycogen instead of 10 g/L
MSVF: medium simulating vaginal fluid
NYCB: New York City III broth
SOM: standard error of the mean
