# Supplementary material for: Glycogen availability and pH variation in a medium simulating vaginal fluid influence the growth of vaginal Lactobacillus species and Gardnerella vaginalis
Source: BMC Microbiol. 2023 Jul 13;23:186. doi: 10.1186/s12866-023-02916-8 (PMC10339506; doi:10.1186/s12866-023-02916-8)
Supplement: Supplementary file 1 — Supplementary Material 1 [file 12866_2023_2916_MOESM1_ESM.docx]

**Supplementary Information**

**Additional file 1**

**Supplementary Methods**

**Supplementary References**

**Additional file 2**

**Table S1** Potential glycogen utilization proteins. *In silico* search of protein database for homologous proteins of *L. jensenii* 62G (ATCC 25282 [DSM 20557]), *L. gasseri* 63 AM (ATCC 33323), *L. crispatus* JV-V01, and *G. vaginalis* JCP8151A

**Additional file 3**

**Table S2** Potential transporters for glycogen degradation products. *In silico* search of protein database for homologous proteins of *L. jensenii* 62G (ATCC 25282 [DSM 20557]), *L. gasseri* 63 AM (ATCC 33323), *L. crispatus* JV-V01, and *G. vaginalis* JCP8151A

**Supplementary Figures**

**Supplementary Fig. S1** Comparison of CFU and pH for strains grown in MSVF: *G. vaginalis* at pH 4.5 **(a)** and 5.0 **(b)**; *L. jensenii* at pH 4.0 **(c)**, 4.5 **(d)**, and 5.0 **(e)**; *L. gasseri* at pH 4.5 **(f)** and 5.0 **(g)**; and *L. crispatus* at pH 5.0 **(h)**.

**Supplementary Fig. S2** Comparison of CFU and pH in MRSB and NYCB: *G. vaginalis* in NYCB at pH 4.5 **(a)** and 5.0 **(b)**; *L. jensenii* in MRSB at pH 4.5 **(c)** and 5.0 **(d)**; *L. gasseri* in MRSB at pH 4.0 **(e)**, 4.5 **(f)**, and 5.0 **(g)**; and *L. crispatus* in MRSB at pH 4.5 **(h)** and pH 5.0 **(i)**.

**Supplementary Fig. S3** Direct comparison of CFU at the same time points when grown in varying amounts of glycogen (10, 5, or 0 g/L) at different pH levels where growth occurred; **(a)** *G. vaginalis*; **(b)** *L. jensenii*; **(c)** *L. crispatus*; and **(d)** *L. gasseri*.

**Supplementary Fig. S4** Changes in the pH of the cultures grown in MSVF with half the amount of glycogen or no glycogen; **(a)** MSVF_5Gly pH 4.5, **(b)** MSVF_5Gly pH 5.0, and **(c)** MSVF_0Gly pH 5.0.

**Supplementary Fig. S5** *G. vaginalis* and *L. jensenii* do not require glucose for growth.

**Supplementary Fig. S6** The *N* terminus of *L. crispatus* JV-V01 is intact, allowing utilization of glycogen.

**Supplementary Methods**

***In silico* search for glycogen utilization and glycogen transport proteins**

First, we searched in the UniProt database (<https://www.uniprot.org/>) for “type” protein among *Lactobacillus* and *Gardnerella* species as specifically as possible. If no protein was retrieved for our specific strains, we used a match from as closely a related species as possible (e.g., other *Lactobacillus* spp., other *Gardnerella* spp., *Corynebacterium* spp., *Streptococcus* spp.). Amino acid sequences for query proteins were obtained from UniProt or from the NCBI protein database (<https://www.ncbi.nlm.nih.gov/protein/>). The protein sequence was entered into blastp (<https://blast.ncbi.nlm.nih.gov/Blast.cgi>) [1] to interrogate the indicated taxa in the non-redundant protein database:

*Lactobacillus jensenii* 62G, ATCC 25282 (aka DSM 20557) (taxid:1423762)

*Lactobacillus gasseri* 63 AM, ATCC 33323 (taxid:324831)

*Lactobacillus crispatus* JV-V01 (taxid:491076)

*Gardnerella vaginalis* JCP8151A (taxid:1261067)

If no 99-100% matching homologous protein was found for one of the strains listed above, the query protein served as the reference protein and results from the blastp were entered into Table S1 if the match reported ≥40% identity or ≥50% similarity and ≥75% coverage and into Table S2 if the match reported ≥50% and ≥75% coverage. If a 99-100% match was found for one of the listed strains, the blastp was repeated using that amino acid sequence and the results entered into the tables using the same criteria for reporting a match. Each protein reported was queried against the NCBI protein database to ensure that the sequence is still valid, and the link was for the correct strain.

Proteins sought were those discussed in the manuscript text – glycogen utilization proteins α-glucosidase, α-amylase, type I pullulanases (including neopullulanase and isoamylase) and type II pullulanases (including amylopullulanase and isopullulanase), glycogen debranching protein, and intracellular maltogenic amylase [2-10]. Transport proteins included MalEFG/MsmK transport operons and MalP, MalH, and PgmB accessory proteins [11] and OmpR family regulatory proteins [12, 13] reported from *Lactobacillus* spp, and MalXFGK, MusEFGK_2_I, and RafEFGK reported from *Gardnerella* spp. [14]. References for these proteins and the programs used are included in Supplementary References following the Supplementary Figures.

**References for Supplementary Materials**

**Links to genomic sequences**

Gene sequences: <https://www.ncbi.nlm.nih.gov/gene/>

*L. gasseri* ATCC 33323: <https://www.ncbi.nlm.nih.gov/nuccore/WBMG01000001.1?from=175734&to=177350&report=gbwithparts>

*L. jensenii* ATCC 25258 (aka DSM20557): <https://www.ncbi.nlm.nih.gov/nuccore/NZ_WKKD01000018.1?from=15773&to=17416&strand=2>

*L. crispatus* JV-V01: <https://www.ncbi.nlm.nih.gov/datasets/genome/?taxon=491076> GCA_000160515.1

*G. vaginalis* JCP8151A: <https://www.ncbi.nlm.nih.gov/datasets/genome/?taxon=1261067> GCA_000414505.1

DNA sequences from which the amino acid sequences for the type I pullulanases of *L. crispatus* shown in Fig. S1 were translated:

RL30, [NKKT01000157.1](https://www.ncbi.nlm.nih.gov/nuccore/NKKT01000157.1)

RL06, [NKLO01000298.1](https://www.ncbi.nlm.nih.gov/nuccore/NKLO01000298.1)

LB63, [JACCPR010000155.1](https://www.ncbi.nlm.nih.gov/nuccore/JACCPR010000155.1)

BN2, [JAOBGO010000136.1](https://www.ncbi.nlm.nih.gov/nuccore/JAOBGO010000136.1)

JV-V01, [ACKR01000196.1](https://www.ncbi.nlm.nih.gov/nuccore/ACKR01000196.1).

**Links for protein sequences**

Glycosidase hydrolase family 13 members: <http://www.cazy.org/GH13.html>

National Center for Biotechnology: <https://www.ncbi.nlm.nih.gov/>

Protein amino acid sequences: <https://www.ncbi.nlm.nih.gov/protein/>

Protein BLAST (blastp): <https://blast.ncbi.nlm.nih.gov/Blast.cgi>

UniProt: <https://www.uniprot.org/>

**References for glycogen utilization and transport proteins and amino acid sequences**

BLAST® NCBI [1]

Amylases, Pullulanases, and other glycogen degrading enzymes [2-10]

Transporters of glycogen breakdown products [11-14]

1. Altschul SF, Madden TL, Schaffer AA, Zhang J, Zhang Z, Miller W, et al. Gapped BLAST and PSI-BLAST: a new generation of protein database search programs. Nucleic Acids Res. 1997;25(17):3389-402; doi: 10.1093/nar/25.17.3389.

2. Bhandari P, Tingley J, Abbott DW, Hill JE. Glycogen degrading activities of catalytic domains of α-amylase and α-amylase-pullulanase enzymes conserved in *Gardnerella* spp. from the vaginal microbiome. J Bacteriol. 2023:e0039322; doi: 10.1101/2022.10.19.512974.

3. Oh KW, Kim MJ, Kim HY, Kim BY, Baik MY, Auh JH, et al. Enzymatic characterization of a maltogenic amylase from *Lactobacillus gasseri* ATCC 33323 expressed in *Escherichia coli*. FEMS Microbiol Lett. 2005;252(1):175-81; doi: 10.1016/j.femsle.2005.08.050.

4. Moller MS, Fredslund F, Majumder A, Nakai H, Poulsen JC, Lo Leggio L, et al. Enzymology and structure of the GH13_31 glucan 1,6-alpha-glucosidase that confers isomaltooligosaccharide utilization in the probiotic *Lactobacillus acidophilus* NCFM. J Bacteriol. 2012;194(16):4249-59; doi: 10.1128/JB.00622-12.

5. Bhandari P, Tingley JP, Palmer DRJ, Abbott DW, Hill JE. Characterization of an alpha-glucosidase enzyme conserved in *Gardnerella* spp. isolated from the human vaginal microbiome. J Bacteriol. 2021;203(17):e0021321; doi: 10.1128/JB.00213-21.

6. Nunn KL, Clair GC, Adkins JN, Engbrecht K, Fillmore T, Forney LJ. Amylases in the human vagina. mSphere. 2020;5(6):e00943-20; doi: 10.1128/mSphere.00943-20.

7. Hertzberger R, May A, Kramer G, van Vondelen I, Molenaar D, Kort R. Genetic elements orchestrating *Lactobacillus crispatus* glycogen metabolism in the vagina. Int J Mol Sci. 2022;23(10):5590; doi: 10.3390/ijms23105590.

8. van der Veer C, Hertzberger RY, Bruisten SM, Tytgat HLP, Swanenburg J, de Kat Angelino-Bart A, et al. Comparative genomics of human *Lactobacillus crispatus* isolates reveals genes for glycosylation and glycogen degradation: implications for *in vivo* dominance of the vaginal microbiota. Microbiome. 2019;7(1):49; doi: 10.1186/s40168-019-0667-9..

9. Woolston BM, Jenkins DJ, Hood-Pishchany MI, Nahoum SR, Balskus EP. Characterization of vaginal microbial enzymes identifies amylopullulanases that support growth of *Lactobacillus crispatus* on glycogen. bioRxiv. 2021:2021.07.19.452977; doi: 10.1101/2021.07.19.452977.

10. Zhang J, Li L, Zhang T, Zhong J. Characterization of a novel type of glycogen-degrading amylopullulanase from *Lactobacillus crispatus*. Appl Microbiol Biotechnol. 2022;106(11):4053-64; doi: 10.1007/s00253-022-11975-2.

11. Ganzle MG, Follador R. Metabolism of oligosaccharides and starch in lactobacilli: a review. Front Microbiol. 2012;3:340; doi: 10.3389/fmicb.2012.00340.

12. Fang FC, Frawley ER, Tapscott T, Vazquez-Torres A. Bacterial stress responses during host infection. Cell Host Microbe. 2016;20(2):133-43; doi: 10.1016/j.chom.2016.07.009.

13. Guffey AA, Loll PJ. 2021. Regulation of resistance in vancomycin-resistant enterococci: The VanRS two-component system. Microorganisms 9.

14. Bhandari P, Hill JE. Transport and utilization of glycogen breakdown products by *Gardnerella* spp. from the human vaginal microbiome. Microbiol Spectr. 2023;11(2):e04435-22; doi: 10.1101/2022.11.01.514706.
