# Supplementary material for: Glycogen availability and pH variation in a medium simulating vaginal fluid influence the growth of vaginal Lactobacillus species and Gardnerella vaginalis
Source: BMC Microbiol. 2023 Jul 13;23:186. doi: 10.1186/s12866-023-02916-8 (PMC10339506; doi:10.1186/s12866-023-02916-8)
Supplement: Supplementary file 2 — Supplementary Material 2 [file 12866_2023_2916_MOESM2_ESM.docx]

**Supplementary Figures**

**
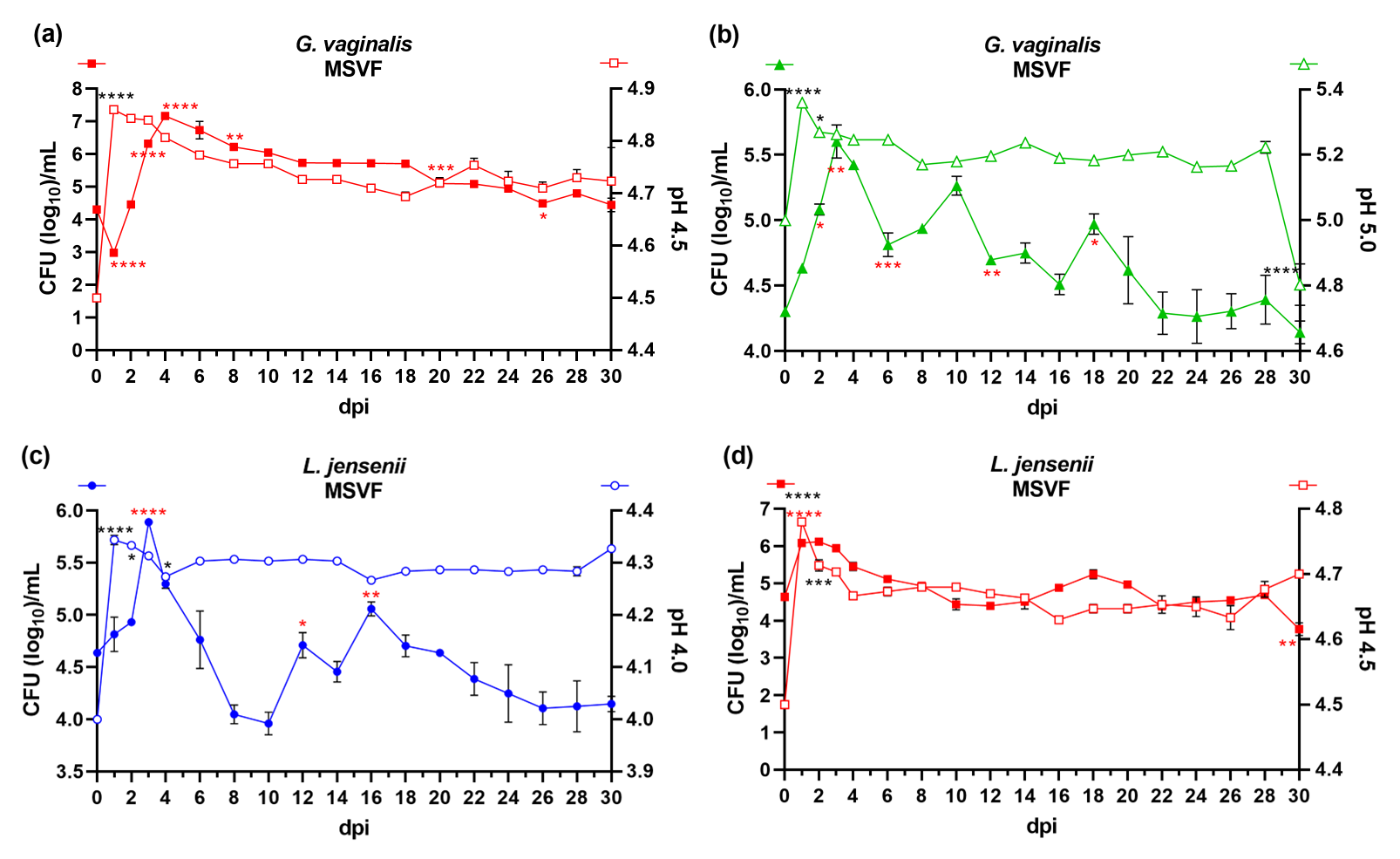
**

**
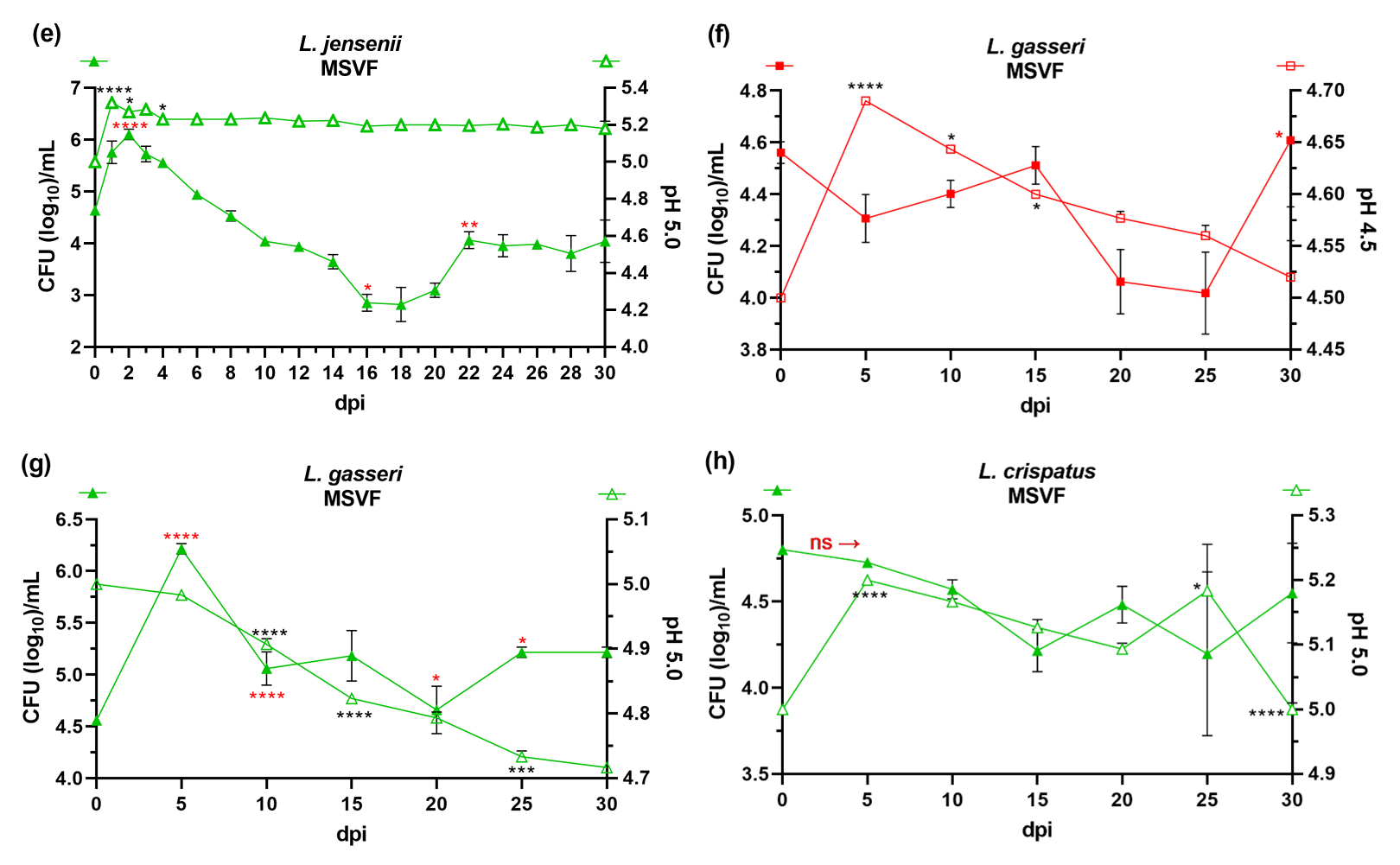
**

**Fig. S1** Comparison of CFU and pH for strains grown in MSVF. Growth patterns of *G. vaginalis* JCP8151A*, L. jensenii* 62G, *L. gasseri* 63 AM, and *L. crispatus* JV-V01 in MSVF and the pH levels of these cultures across time are compared directly in the panels above. Strains were grown overnight in MRSB (lactobacilli) at pH 6.3 or NYCB (*G. vaginalis*) at pH 7.3; cells were pelleted, washed, and resuspended in MSVF at the appropriate pH. One-mL aliquots were pipetted into the wells of a 24-well microtiter plate and inoculated with ~10^4^ CFU. The plates were sealed with breathable membrane and the cultures were incubated at 37°C under 5% CO_2_ in a humid chamber for 30 dpi. Samples were taken at 1-d intervals through 4 dpi and then every 2 d for *L. jensenii* and *G. vaginalis* and at 5-d intervals for *L. gasseri* and *L. crispatus* over the 30-d growth cycle and the CFU/mL and the pH were determined. *G. vaginalis* at pH 4.5 **(a)** and 5.0 **(b)**; *L. jensenii* at pH 4.0 **(c)**, 4.5 **(d)**, and 5.0 **(e)**; *L. gasseri* at pH 4.5 **(f)** and 5.0 **(g)**; and *L. crispatus* at pH 5.0 **(h)**. Each symbol represents the mean of three independent experiments ± SOM. Two-way ANOVA with Tukey’s multiple comparisons posttest was done to determine significant differences between time points across the growth curves. *, *P* <0.05; **, *P* <0.01; ***, *P* <0.001; ****, *P* <0.0001. Red asterisks, CFU/mL; black asterisks, pH

**
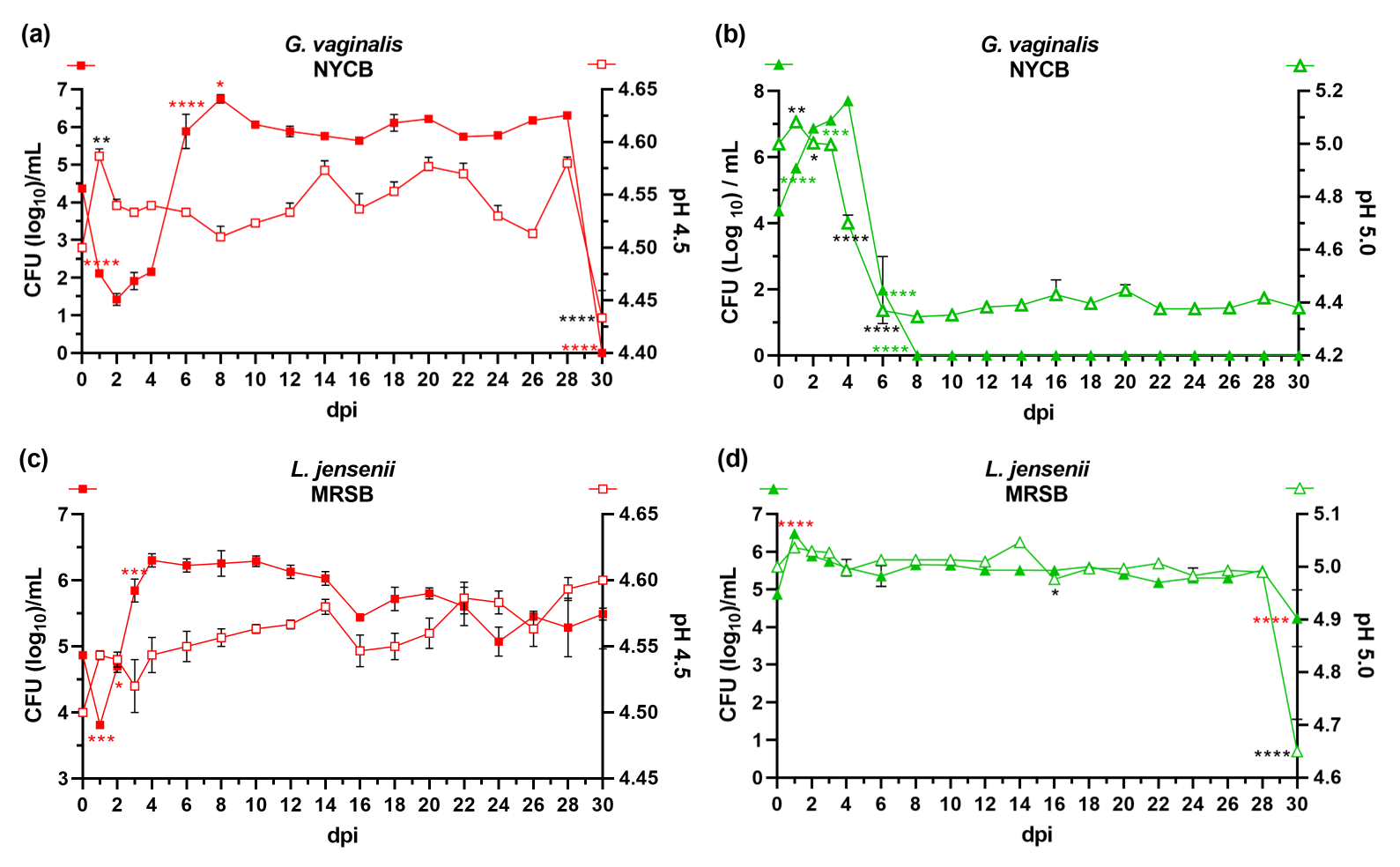
**

**
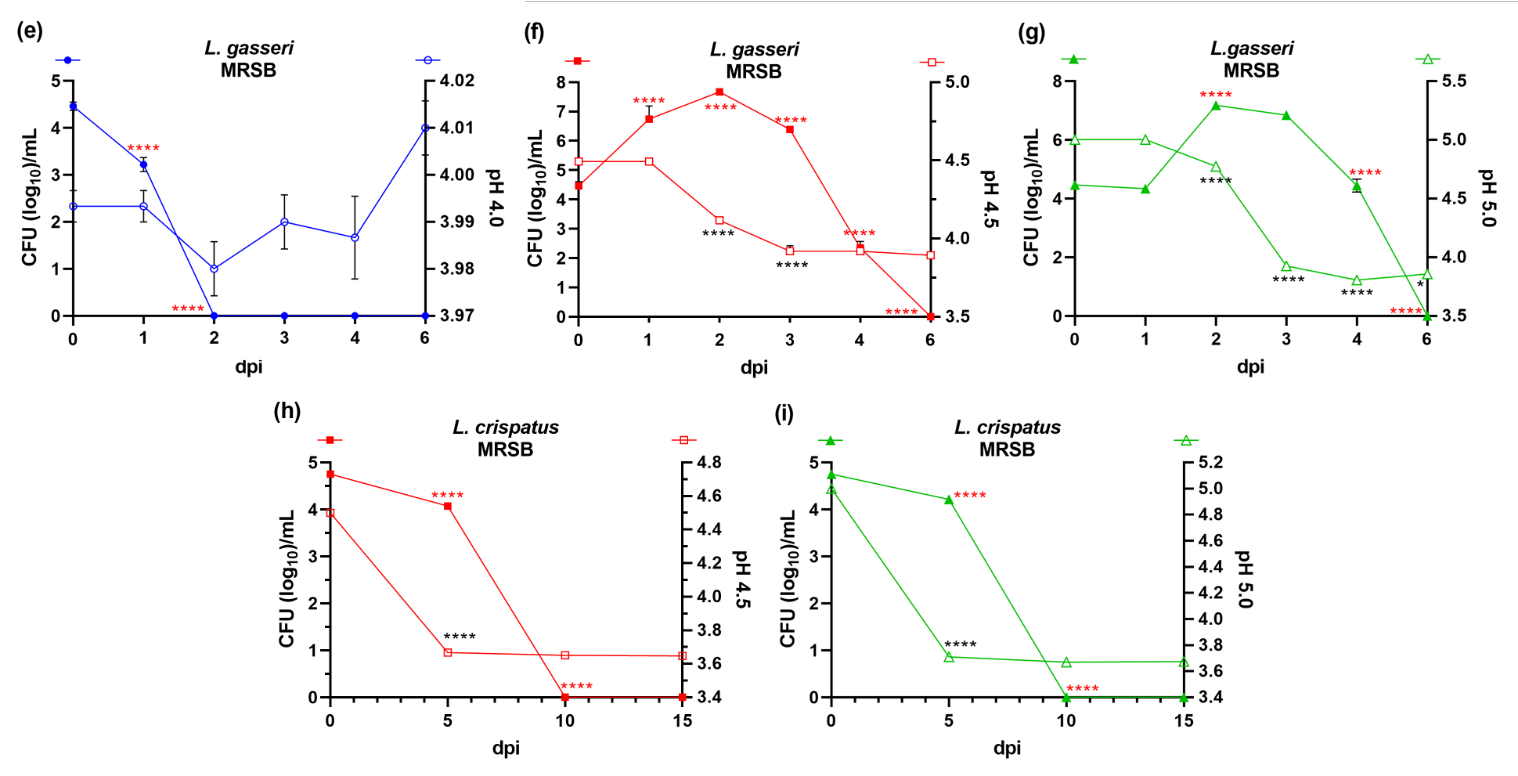
**

**Fig. S2** Growth patterns of *G. vaginalis* JCP8151A in NYCB and *L. jensenii* 62G, *L. gasseri* 63 AM, and *L. crispatus* JV-V01 in MRSB and the pH levels of these cultures across time are compared directly in the panels above. Strains were grown overnight as described in Fig. S1. Cells were pelleted, washed, and resuspended in NYCB for *G. vaginalis* or MRSB for *L. jensenii*, *L. gasseri*, and *L. crispatus* at pH 4.0, pH 4.5, and pH 5.0. Samples were taken over a 30-d growth cycle for *G. vaginalis* and *L. jensenii*, over a 15-d growth cycle for *L. crispatus*, and over 6 d for *L. gasseri* and the CFU/mL were determined. *G. vaginalis* at pH 4.5 **(a)** and 5.0 **(b)**; *L. jensenii* at pH 4.6 **(c**) and 5.0 **(d)**; *L. gasseri* at pH 4.0 **(e)** and 4.5 **(f)** and 5.0 **(g)**; and *L. crispatus* at pH 4.5 **(h)** and 5.0 **(i)**. Each symbol represents the mean of three independent experiments ± SOM. Two-way ANOVA with Tukey’s multiple comparisons posttest was done to determine significant differences between time points across the growth curves. *, *P* <0.05; **, *P* <0.01; ***, *P* <0.001; ****, *P* <0.0001. Red asterisks, CFU/mL; black asterisks, pH

**
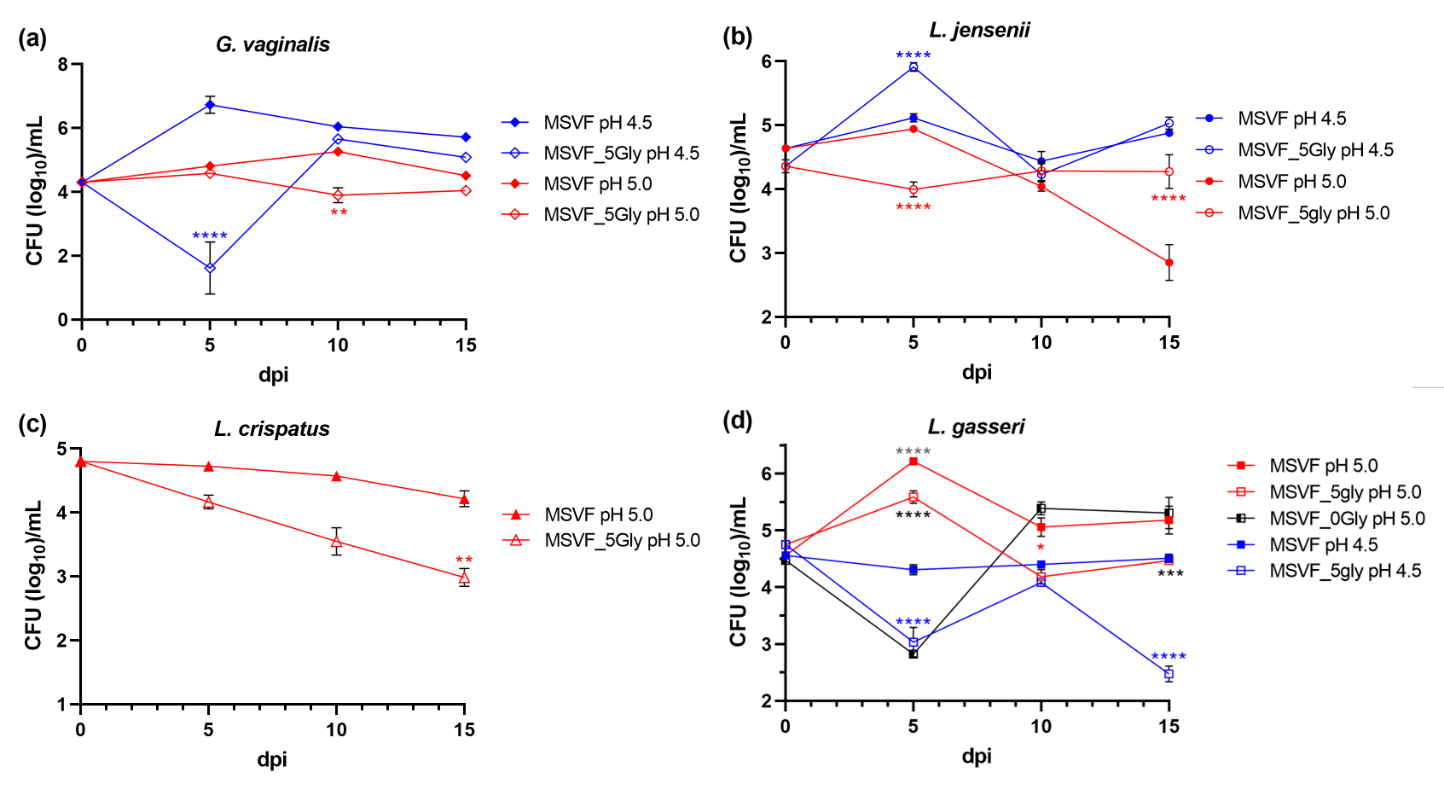
**

**Fig. S3** Direct comparison of CFU at the same time points when grown in varying amounts of glycogen, standard MSVF, MSVF_5Gly, or MSVF_0Gly containing 10 g/L, 5 g/L, or no glycogen, respectively. Only starting pH levels at which growth occurred for 15 dpi are shown on the graphs. Each symbol represents the mean of three independent experiments ± SOM. Two-way ANOVA with Šídák's multiple comparisons test was used to compare CFU at specific time points; significance between MSVF and MSVF_5Gly at pH 4.5 indicated by blue asterisks, between MSVF and MSVF_5Gly at pH 5.0 indicated by red asterisks, between MSVF_0Gly and MSVF_5Gly by black asterisks, and between MSVF_0Gly and MSVF by gray asterisks; *, *P* <0.05; **, *P* <0.01; ***, *P* <0.001; ****, *P* <0.0001

**
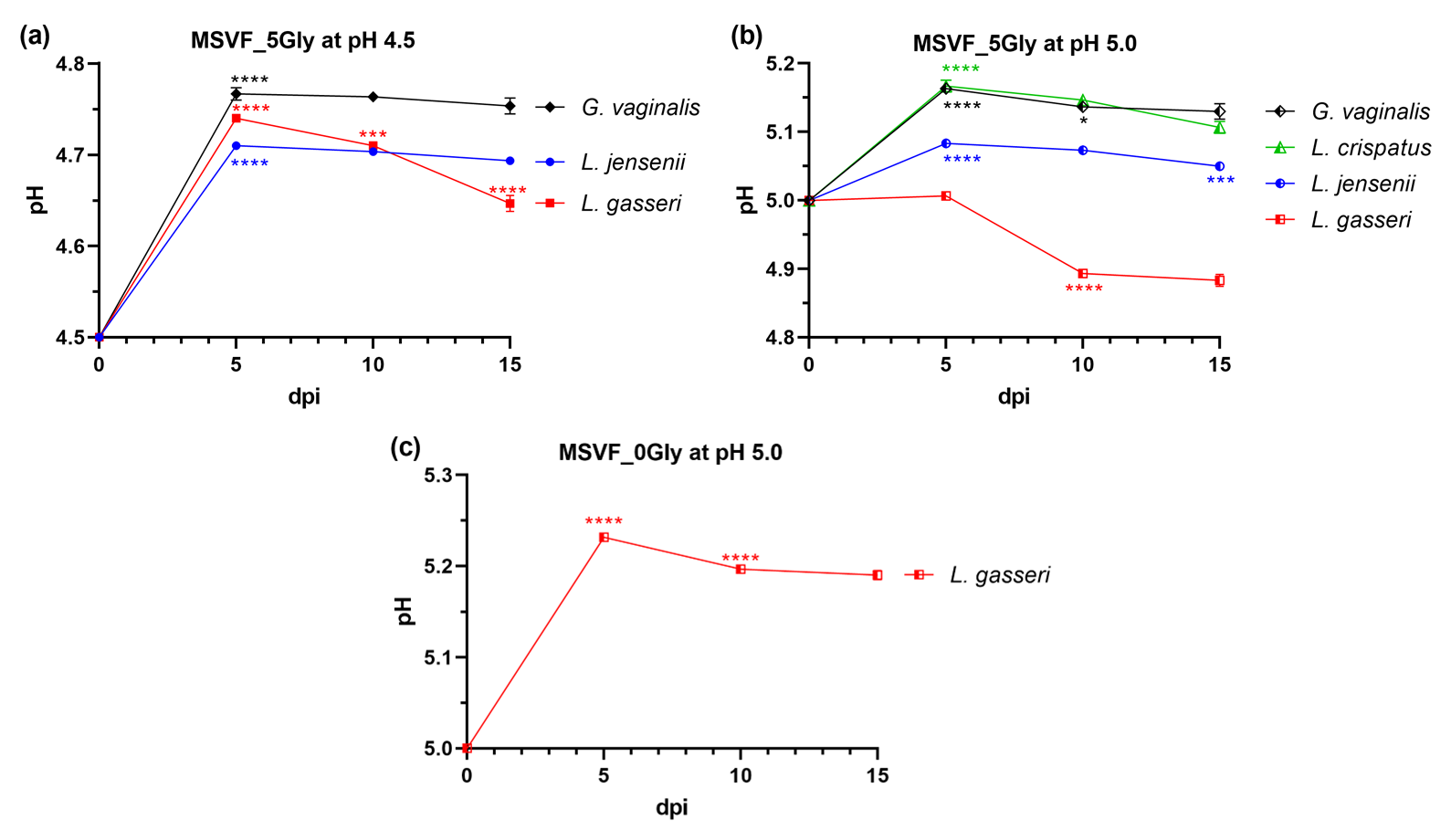
**

**Fig. S4** Changes in the pH of the cultures grown in MSVF with half the amount of glycogen or no glycogen. Strains were grown as described in Fig. S1. The pH of each culture was measured at 5-d intervals over a 15-d growth cycle at **(a)** MSVF_5Gly at pH 4.5, **(b)** MSVF_5Gly at pH 5.0, and **(c)** MSVF_0Gly at pH 5.0. If a strain did not grow at a specific starting pH, the data are not included on the graphs. Each symbol represents the mean of three independent experiments ± SOM. Two-way ANOVA with Tukey’s multiple comparisons posttest was done to determine significant differences between time points across the growth curves; *, *P* <0.05; **, *P* <0.01; ***, *P* <0.001; ****, *P* <0.0001. Black asterisks, *G. vaginalis* JCP8151A; blue asterisks, *L. jensenii* 62G; red asterisks, *L. gasseri* 63 AM; green asterisks, *L. crispatus* JV-V01

**
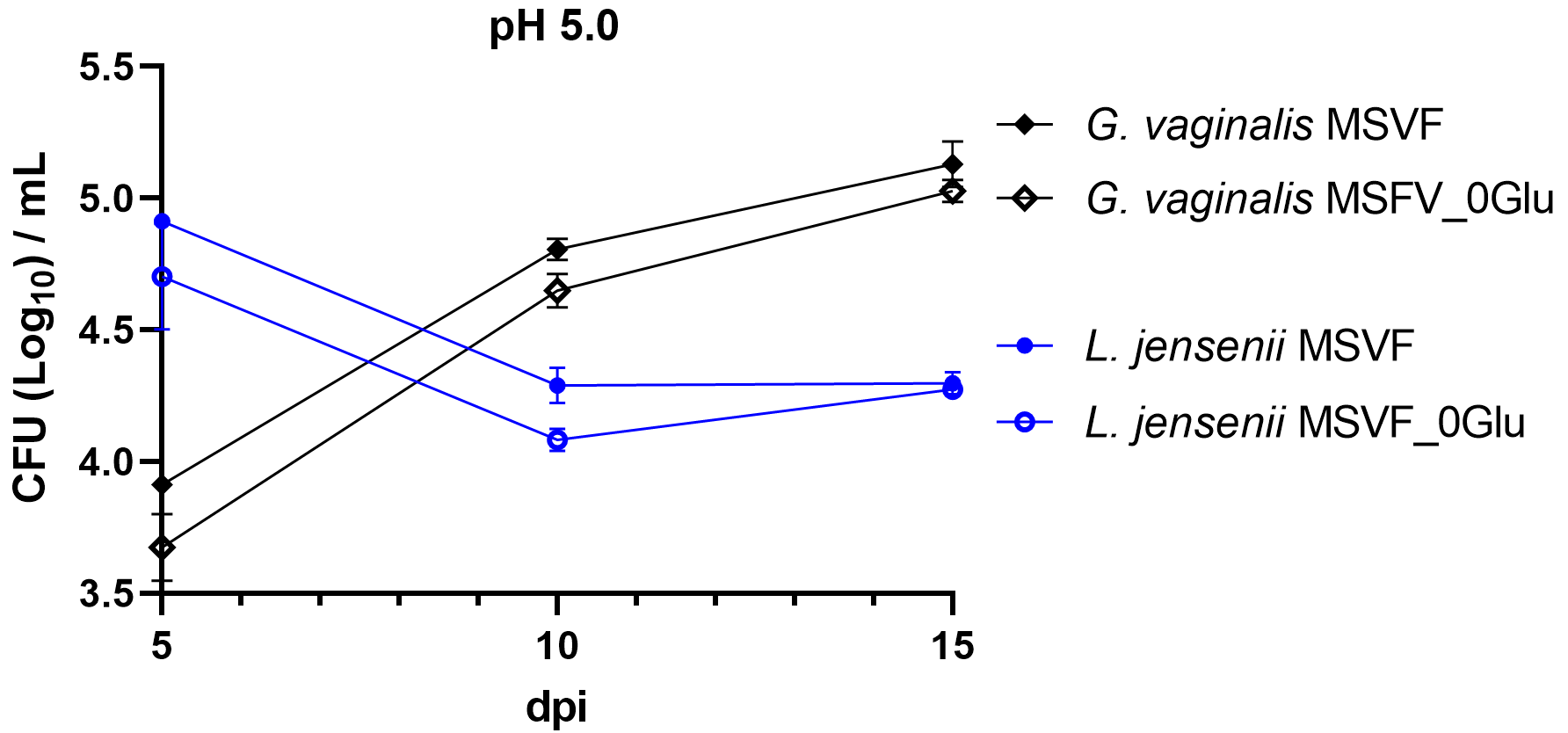
**

**Fig. S5** *G. vaginalis* JCP8151A and *L. jensenii* 62G do not require glucose for growth. Growth in MSVF (containing glycogen and glucose) was compared to MSVF without glucose (MSVF_0Glu). Strains were grown as described in Fig. S1. Samples were taken at 5-d intervals over a 15-d growth cycle and the CFU were determined. Each symbol represents the mean of three independent experiments ± SOM. No significant differences were found between the growth of either strain in MSVF_0Glu compared to MSVF at any time point using two-way ANOVA with Šídák’s multiple comparisons posttest.

RL30 MILWRNLFMNKKSGHNIKFKSIFVCTSAIMSLWLGANLTTTQVHAAEDNAAPKSSEVVGQTNSSKDNAATATVQNQSNAKAKQRQQGVAPQNVPTVLAA [TDM76015.1](https://www.ncbi.nlm.nih.gov/protein/TDM76015.1?report=genbank&log$=prottop&blast_rank=11&RID=6R4A3CHP016)

RL06 M----------------------------MSLWLGANLTTTQVHAAEDNAAPKSSEVVGQTNSSKDNAATATVQNQSNAKAKQRQQGVAPQNVPTVLAA [TDN22590.1](https://www.ncbi.nlm.nih.gov/protein/TDN22590.1)

LB63 M-----------------------------SLWLGANLTTTQVHAAEDNAAPKSSEVVGQTNSSKDNAATATVQNQSNAKAKQRQQGVAPQNVPTVLAA [MBI1715519.1](https://www.ncbi.nlm.nih.gov/protein/MBI1715519.1)

BN2 M--------NKKSGHNIKFKSIFVCTSAIMSLWLGANLTTTQVHAAEDNAAP--------------------------------------QNVPTVLAA [MCT7821720.1](https://www.ncbi.nlm.nih.gov/protein/MCT7821720.1)

**JV-V01** **MILWRNLFMNKKSGHNIKFKSIFVCTSAIMSLWLGANLTTTQVHAAEDNAAPKSSEVVGQTNSSKDNAATATVQNQSNAKAKQRQQGVAPQNVPTVLAA** [**EEJ69081.1**](https://www.ncbi.nlm.nih.gov/protein/EEJ69081.1?report=genbank&log$=prottop&blast_rank=2&RID=6PYGMA0W016)

**Fig. S6** Amino acid alignment of the *N* termini of *L. crispatus* type I pullulanase proteins (PulA). The intact type I pullulanase protein sequence from *L. crispatus* strain RL30 was used to interrogate the non-redundant protein database for *L. crispatus* proteins [1]. Proteins from different strains containing intact amino acid sequences and sequences with deletions in their *N* termini were found. The strains of *L. crispatus* are on the left and accession numbers are on the right. As reported by van der Veer et al., strains such as RL30 with intact *N* termini can utilize glycogen, strains with deletions of the first 32 amino acids utilize glycogen poorly (RL06), and strains with two deletions cannot use glycogen at all (RL05, not sequenced) [1]. JV-V01 type I pullulanase (bold) has an intact *N* terminus and thus was able to utilize glycogen.

1. van der Veer C, Hertzberger RY, Bruisten SM, Tytgat HLP, Swanenburg J, de Kat Angelino-Bart A, et al. Comparative genomics of human *Lactobacillus crispatus* isolates reveals genes for glycosylation and glycogen degradation: implications for *in vivo* dominance of the vaginal microbiota. Microbiome. 2019;7(1):49; doi: 10.1186/s40168-019-0667-9.
